# Sulfated Metabolites of Flavonolignans and 2,3-Dehydroflavonolignans: Preparation and Properties

**DOI:** 10.3390/ijms19082349

**Published:** 2018-08-09

**Authors:** Kateřina Valentová, Kateřina Purchartová, Lenka Rydlová, Lenka Roubalová, David Biedermann, Lucie Petrásková, Alena Křenková, Helena Pelantová, Veronika Holečková-Moravcová, Eva Tesařová, Josef Cvačka, Jiří Vrba, Jitka Ulrichová, Vladimír Křen

**Affiliations:** 1 Institute of Microbiology of the Czech Academy of Sciences, Vídeňská 1083, 14220 Prague, Czech Republic; katerina.purchartova@gmail.com (K.P.); rydlova.l@email.cz (L.R.); biedermann@biomed.cas.cz (D.B.); petraskova@biomed.cas.cz (L.P.); alenka.petrickova@gmail.com (A.K.); pelantova@biomed.cas.cz (H.P.); vholeckov@gmail.com (V.H.-M.); kren@biomed.cas.cz (V.K.); 2 Faculty of Science, Charles University, Department of Physical and Macromolecular Chemistry, Hlavova 2030/8, 12843 Prague, Czech Republic; eva.tesarova@natur.cuni.cz; 3 Department of Medical Chemistry and Biochemistry, Faculty of Medicine and Dentistry, Palacký University, Hněvotínská 3, 77515 Olomouc, Czech Republic; roubalova.lenka@seznam.cz (L.R.); j.vrba@upol.cz (J.V.); jitkaulrichova@seznam.cz (J.U.); 4 Institute of Molecular and Translational Medicine, Faculty of Medicine and Dentistry, Palacký University, Hněvotínská 3, 77515 Olomouc, Czech Republic; 5 Institute of Organic Chemistry and Biochemistry of the Czech Academy of Sciences, Flemingovo nám. 2, 16610 Prague, Czech Republic; josef.cvacka@uochb.cas.cz

**Keywords:** *Silybum marianum*, sulfate, sulfotransferase, biotransformation, metabolites, activity

## Abstract

Silymarin, an extract from milk thistle (*Silybum marianum*) fruits, is consumed in various food supplements. The metabolism of silymarin flavonolignans in mammals is complex, the exact structure of their metabolites still remains partly unclear and standards are not commercially available. This work is focused on the preparation of sulfated metabolites of silymarin flavonolignans. Sulfated flavonolignans were prepared using aryl sulfotransferase from *Desulfitobacterium hafniense* and *p*-nitrophenyl sulfate as a sulfate donor and characterized by high-resolution mass spectrometry (HRMS) and nuclear magnetic resonance (NMR). Their 1,1-diphenyl-2-picrylhydrazyl (DPPH), 2,2′-azinobis-(3-ethylbenzothiazoline-6-sulfonic acid) (ABTS), and *N*,*N*-dimethyl-*p*-phenylenediamine (DMPD) radical scavenging; ferric (FRAP) and Folin–Ciocalteu reagent (FCR) reducing activity; anti-lipoperoxidant potential; and effect on the nuclear erythroid 2-related factor 2 (Nrf2) signaling pathway were examined. Pure silybin A 20-*O*-sulfate, silybin B 20-*O*-sulfate, 2,3-dehydrosilybin-20-*O*-sulfate, 2,3-dehydrosilybin-7,20-di-*O*-sulfate, silychristin-19-*O*-sulfate, 2,3-dehydrosilychristin-19-*O*-sulfate, and silydianin-19-*O*-sulfate were prepared and fully characterized. Sulfated 2,3-dehydroderivatives were more active in FCR and FRAP assays than the parent compounds, and remaining sulfates were less active chemoprotectants. The sulfated flavonolignans obtained can be now used as authentic standards for in vivo metabolic experiments and for further research on their biological activity.

## 1. Introduction

**Figure 1:**
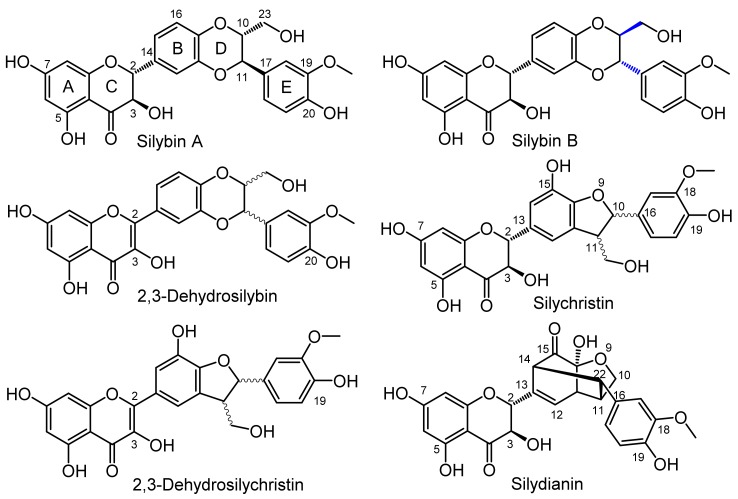
Structures of silymarin flavonolignans used in the study. The structural difference between silybin A and B is shown in blue.

Silymarin is an extract from the fruits of milk thistle (*Silybum marianum* (L.) Gaertn, Asteraceae), a medicinal plant used for various human ailments since the times of the ancient Greeks [1]. Silymarin is a component of a plethora of food supplements and over-the-counter drugs, however the pharmacological properties of its pure constituents have been studied to only a limited extent. Silymarin is a complex mixture of flavonolignans such as silybin A, silybin B, isosilybin, silychristin, silydianin, 2,3-dehydrosilybin, 2,3-dehydrosilychristin and 2,3-dehydrosilydianin (Figure 1), the flavanonol taxifolin, and polymeric phenolics. Silymarin has antioxidant and hepatoprotective effects, as well as anticancer, chemoprotective, dermatoprotective, and hypocholesterolemic activities [2]. Silybin is the major flavonolignan of silymarin, and literature focuses primarily on this compound mainly because of its commercial availability and relatively easy isolation [3]. Although silychristin and silydianin were described more than 40 years ago [4], their biological properties were not investigated until very recently, mainly because of separation problems [5,6]. Even less was known about the 2,3-dehydroflavonolignans [5]. Recently, a new preparatory separation method using Sephadex LH-20 was developed, which provides multigram amounts of pure silychristin A, silydianin, and a fraction containing silybin and isosilybin [7]. Moreover, the methods for the preparation of 2,3-dehydroflavonolignans were optimized and biological and biophysical properties of these compounds, such as antiradical, antilipoperoxidant, and cytoprotective, were recently described by us [5,6].

In recent decades, flavonoids became popular primarily because of their putative antioxidant effects. The antioxidant activity of polyphenolic compounds may be due to antiradical action, inhibition of pro-oxidant enzymes, and/or activation of the nuclear erythroid 2-related factor 2 (Nrf2), which regulates the expression of many antioxidant enzymes [8,9]. Accordingly, as the dietary and supplemental intake of flavonoids increased, their pharmacokinetics and metabolism are now extensively studied [10,11]. Flavonolignans, like other flavonoids, are typically metabolized by phase II biotransformation enzymes; they are sulfated, glucuronidated, or methylated both ex vivo [12] and in vivo [10,13,14]. The biotransformation enzymes exist as isoforms with diverse activity and specificity, and thus can contribute to a large inter- and intra-individual variation in the bioavailability of parent compounds and their metabolites [15,16]. Correct metabolic analysis of blood plasma or urine is practically impossible without using the authentic standards of individual metabolites. To date, the sulfates of silybin and isosilybin were prepared in a quantity sufficient for their detailed structural identification [17]. In this work, we focused on the preparation of structurally characterized sulfated metabolites of pure silybins A and B and of other silymarin flavonolignans, that is, 2,3-dehydrosilybin, silychristin, 2,3-dehydrosilychristin, and silydianin. Moreover, the antioxidant and Nrf2 modulating activities of the obtained flavonolignan sulfates were evaluated using different in vitro assays.

## 2. Results and Discussion

Silymarin is a complex mixture and to study its pharmacokinetics and toxicological properties, authentic standards of all possible metabolites of its components are required. Recently reported sulfates of silybins A and B and of isosilybins A and B were obtained using bacterial aryl sulfotransferase from *Desulfitobacterium hafniense* (AST DH), which selectively catalyzed C-20 OH sulfation of the parent compounds [17]. On the other hand, aryl sulfotransferase IV from rat liver (AST IV) heterologously expressed in *E. coli*, used for the same reactions, exhibited a strict stereoselective sulfating reaction of silybin isomers; silybin B was sulfated yielding silybin B 20-*O*-sulfate, whereas silybin A proved to be completely resistant to the sulfating reaction [18]. In the present work, we focused on the sulfation of six flavonolignans of silymarin, namely silybin A, silybin B, 2,3-dehydrosilybin, silychristin, 2,3-dehydrosilychristin, and silydianin.

### 2.1. Sulfation of Silymarin Flavonolignans by Aryl Sulfotransferase from Rat Liver (AST IV)

Silybin (natural mixture of diastereomers A and B), silychristin, and silydianin were sulfated using whole *E. coli* cells transfected with and overexpressing the gene for AST IV [18]. In accordance with our previous study, efficient sulfation was only found for silybin B (up to 48%, Table 1) with the formation of silybin B 20-*O*-sulfate [18]. When silychristin or silydianin were tested as substrates in this AST IV catalyzed reaction, both consumption of the sulfate donor *p*-nitrophenyl sulfate (*p*-NPS) and formation of *p*-nitrophenol (*p*-NP) were observed (Table 1), which clearly proved the sulfation reactions. However, no sulfated products were isolated and even recovery of the starting material was impossible. We assume that these substrates were probably sulfated, but immediately metabolized by the cells; hence, the sulfation products could not be isolated.

### 2.2. Sulfation of Silymarin Flavonolignans with Aryl Sulfotransferase from Desulfitobacterium hafniense

Compared with our previous work with purified AST DH enzyme [17], the crude extract [19] used here displayed enhanced sulfation activity, and thus substantially reduced reaction time (from several days to *ca* 4–6 h) reaching nearly quantitative conversion in the case of silybin. Silybin A 20-*O*-sulfate and silybin B 20-*O*-sulfate were obtained using this modified procedure in 56% and 40% yield, respectively.

**Figure 2:**
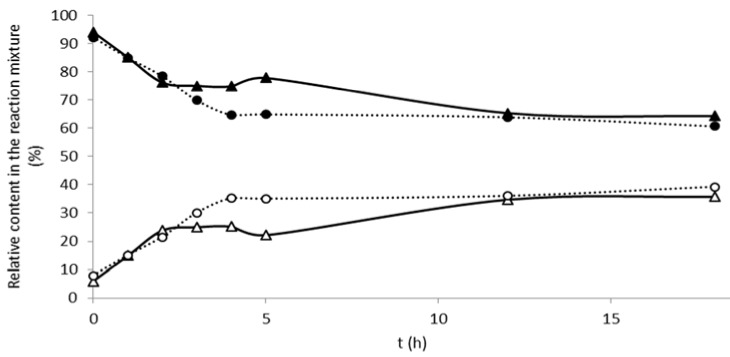
Time course of 2,3-dehydrosilybin sulfation by aryl sulfotransferase (AST) from *D. hafniense*. Both enantiomers were incubated separately with the enzyme and *p*-nitrophenyl sulfate (*p*-NPS) as sulfate donor as described in the Experimental. Relative content of the acceptors, i.e., 2,3-dehydrosilybin A (▲) or 2,3-dehydrosilybin B (●) and respective products, i.e., 2,3-dehydrosilybin A 20-*O*-sulfate (△) or 2,3-dehydrosilybin B 20-*O*-sulfate (○) were determined from the area under the curve (AUC)% from the respective chromatograms.

The time-course of 2,3-dehydrosilybin sulfation was followed separately for enantiomers A and B for 18 h. Sulfated metabolites start to form immediately after the enzyme addition with linear increase of the sulfate concentration within the first five hours and 2,3-dehydrosilybin B was sulfated slightly more quickly than the enantiomer A (Figure 2). The sulfation was much less efficient for this oxidized silybin derivative than for silybin, with an overall yield *ca* 20% (and slightly higher for 2,3-dehydrosilybin A compared with B) and after 4 h reaction, the conversion typically remained under 50%. This is probably caused by the rather different 3D structure of 2,3-dehydrosilybin (planar) compared with silybin (curved) [20] and lower solubility in the reaction mixture. In the case of 2,3-dehydrosilybin, a considerable amount of disulfated product at positions C-7 and C-20 was also isolated in the comparable yields to that of the monosulfates (11.6 vs. 9.4%). We assume that 2,3-dehydrosilybin monosulfate, because of its higher water solubility, competed with 2,3-dehydrosilybin for the (further) sulfation reaction.

**Figure 3:**
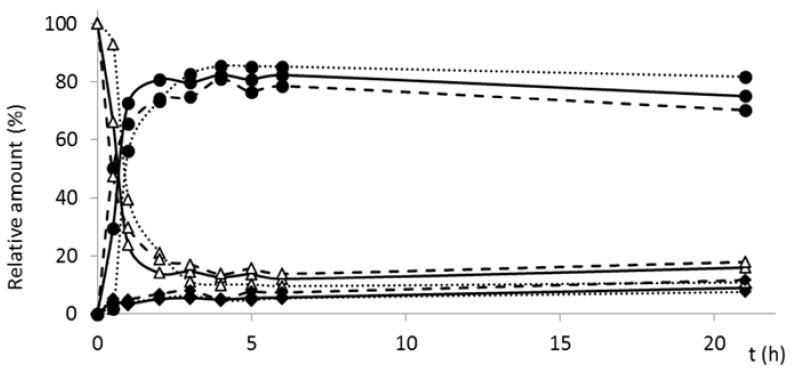
Time-course of silychristin sulfation by AST from *D. hafniense*. The reaction mixtures were incubated with the enzyme and *p*-NPS as sulfate donor separately under Ar (solid line), in low oxygen environment (dashed), and under 100% O_2_ (dotted line) as described in the Experimental. Relative content of silychristin (△), silychristin-19-*O*-sulfate (●), and 2,3-dehydrosilychristin-19-*O*-sulfate (♦) was determined from the AUC% from the respective HPLC chromatograms using the calibration curves of silychristin and 2,3-dehydrosilychristin.

Sulfation of silychristin yielded the major product, silychristin-19-*O*-sulfate, in *ca* 32% yield. Under the alkaline pH of the reaction mixture, silymarin flavonolignans were apt to an air oxidation, thus forming respective 2,3-dehydroderivatives and their sulfates. Oxidation can be limited using inert atmosphere (Ar) and short reaction time [19]. However, in the case of silychristin, 2,3-dehydrosilychristin-19-*O*-sulfate was always formed even under Ar atmosphere (as observed previously with oxidation of silybin and isosilybin [21]). Formation of trace amounts of 2,3-dehydrosilychristin and of disulfates of both silychristin and 2,3-dehydrosilychristin was also observed (confirmed by mass spectrometry (MS) only). In order to determine the effect of oxygen on the formation of 2,3-dehydrosilychristin-19-*O*-sulfate, a kinetic study was performed in the presence of different concentrations of oxygen. The formation of 2,3-dehydrosilychristin-19-*O*-sulfate occurred regardless of the oxygen presence in the reaction mixture (Figure 3). On the other hand, when we tried to directly sulfate 2,3-dehydrosilychristin, no product was formed (not shown). In order to obtain sufficient amounts of 2,3-dehydrosilychristin-19-*O*-sulfate for further experiments, this compound was prepared by the oxidation of silychristin-19-*O*-sulfate by O_2_ in the presence of 4-dimethylaminopyridine (DMAP) with 55% yield.

Sulfation of silydianin yielded a major, but extremely unstable product identified (NMR, MS) as silydianin-19-*O*-sulfate. It quickly decomposed into a mixture of at least five products and, therefore, it was not studied further.

### 2.3. Analytical Characteristics and Structure Elucidation of the Sulfated Metabolites

**Figure 4:**
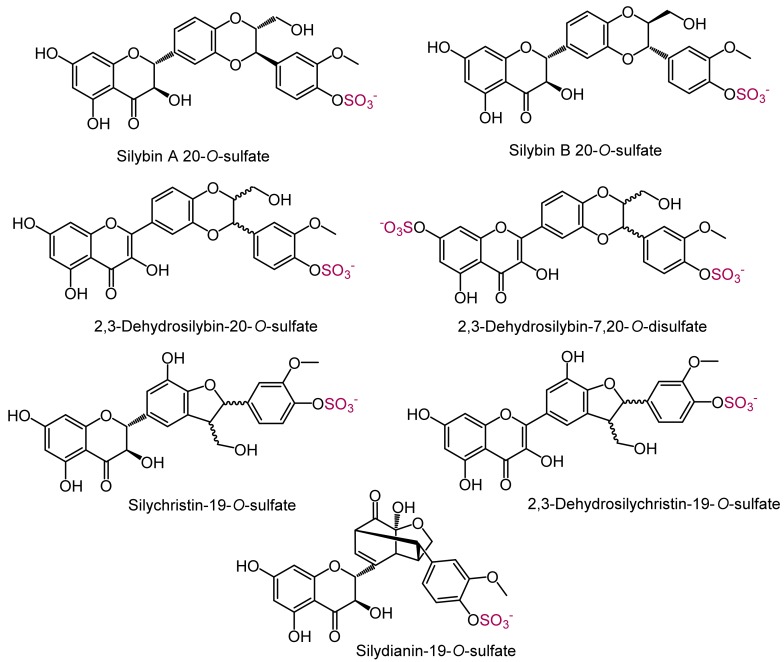
Isolated sulfated products. The sulfate groups are highlighted in red.

Sulfated (2,3-dehydro)flavonolignans (Figure 4) were obtained in isolated yields ranging from 10 to 58%. As a side product of silychristin sulfation, we also isolated 2,3-dehydrosilychristin-19-*O*-sulfate (0.9% yield); nonetheless, this compound was then prepared by silychristin-19-*O*-sulfate oxidation by air in 55% yield. Overall, tens to hundreds of milligrams of all sulfated metabolites were isolated and fully characterized by HPLC, UV, HRMS, and complete NMR spectra (Table 2). The sulfates displayed quite broad tailing peaks in HPLC chromatograms (Figures S1, S2, S5, S7, S10, and S12 in the Supplementary data) because of their amphiphilic properties. The homogeneity of HPLC peaks was assessed by UV spectra scanning of the peaks, and the purity of the compounds was unequivocally confirmed by rigorous analysis of their ^1^H and ^13^C NMR spectra.

NMR spectra of all compounds showed several common structural features: AB system of *meta*–disposed aromatic protons (ring A) and ABC system of 1,3,4-trisubstituted aromatic ring E. The assignment of all individual proton spin systems was achieved by correlation spectroscopy (COSY), and then transferred to carbons by heteronuclear single-quantum correlation spectroscopy (HSQC). Heteronuclear multiple-bond correlation spectroscopy (HMBC) experiment enabled us to assign quaternary carbons and combine partial structures together. The sulfation of individual compounds was confirmed by typical changes of carbon chemical shifts compared with parent compounds: up-field shifted signal of the sulfate-linked carbon (C-7 in the ring A; C-19 or C-20 in the ring E), down-field shifted signals of carbons adjacent to the position of sulfation. Observed changes are consistent with those previously published for quercetin sulfates [19].

### 2.4. The Effect of Sulfation on Radical Scavenging and Anti-Lipoperoxidant Activity of the Flavonolignans

Phase II metabolites are primarily produced in the human body to accelerate the excretion of xenobiotics to the bile and urine. However, in case of various polyphenols, the conjugated metabolites rather than the parent compounds are present in blood, and hence the conjugates may be responsible for some biological effects [15,22]. Therefore, to determine the effect of sulfation on the biological properties of the metabolites, all the isolated sulfates (Figure 4) were tested in several radical scavenging activity assays and compared with their parent molecules. Although most of the tests used are now considered to be rather artificial and with limited relevance for biological systems, they are very useful as first line screening for new compounds and their comparison.

In 1,1-diphenyl-2-picrylhydrazyl (DPPH) scavenging (results are presented as IC_50_ values, which are inversely proportional to radical scavenging activity), 2,3-dehydrosilychristin monosulfate was the most efficient compound (IC_50_ = 8 µM) and slightly better than 2,3-dehydrosilychristin. Similarly, in the case of 2,3-dehydrosilybin, the monosulfate was as active as the parent compound (IC_50_ = 13–14 µM). On the other hand, 2,3-dehydrosilybin-7,20-di-*O*-sulfate, silychristin-19-*O*-sulfate, silybin A, and silybin B were much less active (IC_50_ reached physiologically non-achievable values of 106 µM, 587 µM, 490 µM, and 546 µM, respectively). In the case of silybin sulfates, IC_50_ was higher than the highest concentration tested (i.e., 2500 µM). In 2,2′-azinobis-(3-ethylbenzothiazoline-6-sulfonic acid) cation (ABTS^+^) radical scavenging assay, silychristin was the most active (1.50 vitamin C equivalents, CE) and the sulfated metabolites were always at least slightly less effective than the parent compounds, both silybin sulfates were the least active (0.020 and 0.036 CE). Folin–Ciocalteu reagent (FCR) reducing potential was the highest in the case of 2,3-dehydrosilychristin with 1.58 gallic acid equivalents (GAE), followed by 2,3-dehydrosilybin (1.50 GAE). All sulfates were less active than the parent flavonolignans, with silybin sulfates being the least potent (A 0.08 and B 0.16 GAE). In contrast, the sulfates of 2,3-dehydroflavonolignans tended to be more active in ferric reducing antioxidant power (FRAP) assay; 2,3-dehydrosilybin-7,20-di-*O*-sulfate was the most active with 1.61 equivalents of Fe^2+^, compared to 0.81 eq for the parent compound. High activity of the sulfates was also observed in *N*,*N*-dimethyl-*p*-phenylenediamine (DMPD^+^) radical scavenging assay; 2,3-dehydrosilybin disulfate was the most active (1.90 CE), followed by silychristin and its monosulfate (1.5 CE, Table 3).

Inhibition of lipid peroxidation followed the similar trend as DPPH scavenging, and the most active was 2,3-dehydrosilybin (IC_50_ = 10.4 µM). The more hydrophilic (log *p* < 0) sulfates were less active than the parent compounds, with the exception of 2,3-dehydrosilychristin, whose activity was similar to its sulfate despite their different log *p* (Table 4), probably because of different positioning in the membrane, as in the case of quercetin sulfates [23].

Radical scavenging activity of sulfated flavonolignans is presented here for the first time. The activities of non-sulfated (2,3-dehydro) flavonolignans are mostly in good accordance with those published previously [5,6,20]. Such a complex panel of various antioxidant assays was also used for the first time for optically pure silybins A and B. Surprisingly, significant difference in DPPH scavenging, FRAP, and inhibition of lipid peroxidation activity was found for the diastereomers, with silybin A being always at least slightly more active (Table 3). This is in contrast with the theoretical O–H bond dissociation enthalpies, which are almost the same for both diastereomers [20]. In addition, our results show that the antioxidant/antiradical activities of both sulfated and parent [5] flavonolignans are lower than that of the flavanonol taxifolin (for DPPH scavenging, IC_50_ = 6–10 µM) [24,25], which accounts for up to 5% in various silymarin preparations [26].

### 2.5. The Effect of the Sulfated Metabolites on the Nrf2 Pathway

**Figure 5:**
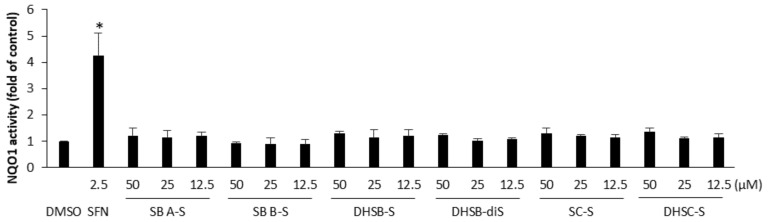
Effect of tested sulfated flavonolignans on NAD(P)/quinone oxidoreductase 1 (NQO1) activity in Hepa1c1c7 cells. Cells were treated for 48 h with 0.1% dimethylsulfoxide (DMSO) (control); 2.5 μM sulforaphane (SFN; positive control); or with indicated concentrations of silybin A 20-*O*-sulfate (SB A-S), silybin B 20-*O*-sulfate (SB B-S), 2,3-dehydrosilybin-20-*O*-sulfate (DHSB-S), 2,3-dehydrosilybin-7,20-di-*O*-sulfate (DHSB-diS), silychristin-19-*O*-sulfate (SC-S), or 2,3-dehydrosilychristin-19-*O*-sulfate (DHSC-S). After treatment, the activity of NQO1 was determined using the NQO1 assay. Data are means ± standard deviation of three experiments. *p* < 0.05 (*) significantly increased versus control.

Another mechanism through which polyphenols may exert their antioxidant action is the activation of the transcription factor Nrf2, which regulates the expression of various cytoprotective antioxidant enzymes, including NAD(P)/quinone oxidoreductase 1 (NQO1) [27]. To examine whether the sulfated flavonolignans activate the Nrf2 pathway, we evaluated the changes in the activity of NQO1 in murine hepatoma Hepa1c1c7 cells. Cell treatment for 48 h with 2.5 µM sulforaphane, a classical Nrf2 activator, the positive control [28], resulted in a 4.3-fold increase in the activity of NQO1 compared with the control. In contrast, flavonolignan sulfates at non-cytotoxic concentrations had only a mild or negligible effect on the NQO1 activity in Hepa1c1c7 cells (Figure 5). At the highest concentration tested (50 µM) and after 48 h of treatment, the activity of NQO1 reached 1.2-fold by silybin A 20-*O*-sulfate, 0.9-fold by silybin B 20-*O*-sulfate, 1.3-fold by 2,3-dehydrosilybin-20-*O*-sulfate, 1.2-fold by 2,3-dehydrosilybin-7,20-di-*O*-sulfate, 1.3-fold by silychristin-19-*O*-sulfate, and 1.4-fold by 2,3-dehydrosilychristin-19-*O*-sulfate. It may be mentioned that the effect of non-sulfated flavonolignans on the Nrf2 pathway was also recently examined in Hepa1c1c7 cells, but 2,3-dehydrosilydianin was only identified as an Nrf2 activator [29].

## 3. Materials and Methods 

### 3.1. General Methods 

#### 3.1.1. NMR 

NMR spectra were recorded on a Bruker Avance III 600 MHz spectrometer (600.23 MHz for ^1^H, 150.94 MHz for ^13^C) at 30 °C in dimethylsulfoxide (DMSO)-*d*_6_. Residual solvent signal (*δ*_H_ 2.500 ppm, *δ*_C_ 39.60 ppm) served as an internal standard. NMR experiments: ^1^H NMR, ^13^C NMR, gCOSY, gHSQC, and gHMBC were performed using the standard manufacturer’s software. ^1^H NMR and ^13^C NMR spectra were zero filled to four-fold data points and multiplied by window function before Fourier transformation. A two-parameter double-exponential Lorentz–Gauss function was applied for ^1^H to improve resolution, and line broadening (1 Hz) was applied to get better ^13^C signal-to-noise ratio. Chemical shifts are given in *δ*-scale with digital resolution justifying the reported values to three (*δ*_H_) or two (*δ*_C_) decimal places.

#### 3.1.2. Mass Spectrometry (MS)

Mass spectra in the negative ion mode were measured using LTQ Orbitrap XL hybrid mass spectrometer (Thermo Fisher Scientific, Waltham, MA, USA) equipped with an electrospray ion source. The samples were dissolved in MeOH and introduced into the mobile phase flow (MeOH/H_2_O 4:1; 100 µL/min) using a 2 µL loop. Spray voltage, capillary voltage, tube lens voltage, and capillary temperature were 4.0 kV, −16 V, −120 V, and 275 °C, respectively.

#### 3.1.3. Analytical HPLC-PDA

All analytical HPLC analyses were performed using the Shimadzu Prominence System (Shimadzu, Kyoto, Japan) consisting of a DGU-20A mobile phase degasser, two LC-20AD solvent delivery units, a SIL-20AC cooling auto sampler, a CTO-10AS column oven, and an SPD-M20A diode array detector. Chromatographic data and UV spectra were collected and processed using Shimadzu Solution software (ver. 5.75 SP 2; Shimadzu, Kyoto, Japan) at a rate of 40 Hz. 

Chromolith Diol (50 × 4.6 mm i.d.) or chromolith performance RP-18e monolithic column (100 × 3 mm i.d., both Merck, Darmstadt, Germany) coupled with a guard column (5 × 4.6 mm i.d., Merck, DE) were used for the separation of silybin sulfates and all remaining sulfates, respectively. Gradient elution using mobile phase A: MeCN/H_2_O/HCO_2_H (5/95/0.1; *v*/*v*/*v*) and mobile phase B: MeCN/H_2_O/HCO_2_H (80/20/0.1; *v*/*v*/*v*) were employed. Gradient G1 (silybin): 0–2 min 0% B, 2–7 min 0–90% B, 7–8 min 90% B, 8–11 min 90–0% B, 14 min stop; gradient G2 (2,3-dehydrosilybin): 0–6 min 20% B, 6–10 min 40% B, 10–11 min 80% B, 11–13 min 20% B, 13 min 0% B; gradient G3 (silychristin, silydianin, and 2,3-dehydrosilychristin): 0–5 min 0% B, 5–8 min 25% B, 8–10 min 60% B, 11–12 min 0% B. The flow rate was 2 mL/min for G1 or 1.2 mL/min for G2 and G3 at 25 °C for all analyses. The PDA data were acquired in the 200–450 nm range and 285 nm (silybin, silychristin, silydianin) or 360 nm (2,3-dehydrosilybin, 2,3-dehydrosilychristin derivatives) signals were extracted.

### 3.2. Preparation of the Flavonolignans 

Silymarin was purchased from Liaoning Senrong Pharmaceutical (Panjin, China, batch No. 120501). Silybin [3] was isolated by the standard method. Briefly, silymarin was suspended in MeOH and after brief stirring, undissolved solid was filtered out and washed with MeOH, giving silybin of 95% purity. Optically pure silybin A and silybin B were prepared by enzymatic discrimination as described previously [30]. From silybin, 2,3-dehydrosilybin was prepared as previously described [31]. Silychristin and silydianin were isolated from silymarin as published previously [6,7]. Briefly, silymarin void of silybin was injected on the Sephadex LH-20 gel column and eluted with MeOH at 40 °C to obtain silydianin, which was purified by crystallization (MeOH), and silychristin that was re-injected to the same column to yield silychristin of the purity >95%. According to the literature [5], 2,3-dehydrosilychristin was prepared by oxidation of silychristin.

### 3.3. Sulfation of the Flavonolignans by Aryl Sulfotransferase from Rat Liver (AST IV)

The plasmid bearing *astIV* gene was kindly provided by Prof. L. Elling, RWTH Aachen, Germany. The enzyme was expressed in *E. coli* BL21 (DE3) Gold as described [18], with modifications reported in our previous work [19]. Whole *E. coli* cells producing the enzyme, freshly prepared according to the literature [19], were employed in the biotransformation experiments as a catalyst.

Silybin, silybin A, silybin B, silychristin, and silydianin (separately, each 50 mg, 0.104 mmol) and *p*-nitrophenyl sulfate (*p*-NPS, 35 mg, 0.136 mmol; Sigma-Aldrich, St. Louis, MO, USA) were dissolved in 3 mL DMSO, and 2 g (wet based) of whole cells expressing AST IV resuspended in K-phosphate buffer (pH 7.5, 17 mL) were added. The reaction mixture was incubated on a rotary shaker at 37 °C for 24 h. The cells were then removed by centrifugation (30 min, 5000 rpm, 5404 R Eppendorf, Germany), a new portion of fresh cells (2 g in 15 mL, wet based, resuspended in K-phosphate buffer) were added to the supernatant and incubated for 24 h. This step was repeated once more with a total reaction time of 72 h. The reaction was monitored by HPLC and after 72 h, the reaction mixtures, containing the sulfated product(s), were purified by preparative HPLC employing Asahipak GS-310 20F (Shodex, Tokyo, Japan) column with isocratic elution with 100% methanol. The fraction containing the putative sulfated product(s) was evaporated, dissolved in 1 mL of 80% methanol, and loaded onto a Sephadex LH-20 column (15 g dry weight, 12 mm i.d.) packed and eluted with 80% methanol. Only one sulfated product was obtained; silybin B 20-*O*-sulfate (14.5 mg, 0.03 mmol, yield 25%), whose identity was confirmed by comparison with previously published data [18].

### 3.4. Preparation of Sulfated Flavonolignans by Aryl Sulfotransferase from Desulfitobacterium hafniense (AST DH)

The AST DH enzyme was expressed as described by van der Horst et al. 2012 [32] with amendments described in detail in our previous work [19]. The crude enzyme was always prepared fresh, standard enzymatic assays were performed at 30 °C and AST DH specific activity was ≥25,000 U/mg.

#### 3.4.1. Silybin

Silybin A and B diastereomers (separately, each 200 mg, 0.415 mmol) were dissolved in 5 mL of acetone. Then, 24 mL of 100 mM Tris-glycine buffer (pH 8.9), *p*-NPS (125 mg, 0.43 mmol in 5 mL of the Tris–glycine buffer), and 2 mL of AST DH (4340 U/mL in the same buffer) were added to the substrate solution and the mixture was incubated at 30 °C. The reaction progress was monitored by HPLC and after 4 h, the conversion was typically over 90%. The reaction mixture volume was halved by evaporation in vacuo to remove all organic solvents, pH was adjusted to 7.5–7.7, and *p*-nitrophenol (*p*-NP) and residual starting material were extracted (3 × 50 mL EtOAc). The aqueous phase (15 mL) containing the sulfated products was evaporated, the residue was dissolved in 2 mL of 80% methanol and loaded onto a Sephadex LH-20 column (30 g dry weight, 3 cm i.d.) packed and equilibrated with mobile phase MeOH/H_2_O (8/2; *v*/*v*). The fractions were analyzed by TLC (EtOAc/MeOH/HCO_2_H, 4/1/0.01; *v*/*v*/*v*) and the fractions containing the respective product were combined and evaporated in vacuo at 45 °C. Both sulfates were obtained as yellowish solids, silybin A-20-*O*-sulfate (131 mg, 56% yield, and purity 99%, Figure S1, Supplementary Material), and silybin B 20-*O*-sulfate (93 mg, 40% yield, purity 99.9%, Figure S2, Supplementary Material). Their identities were confirmed by comparison of their HPLC profile, UV, MS, and NMR spectra with previously published ones [17]. 

#### 3.4.2. 2,3-Dehydrosilybin

The time course of 2,3-dehydrosilybin sulfation was followed in a kinetic study. For this purpose, 50 mg of 2,3-dehydrosilybin (A and B enantiomers separately, 0.104 mmol) suspended in 1.25 mL of acetone was mixed with *p*-NPS (32 mg, 0.108 mmol, 1 mL), 5.75 mL of the Tris-glycine buffer, and 1 mL of AST DH (4340 U/mL). The reaction mixture was incubated under Ar atmosphere at 30 °C, 2,3-dehydrosilybin dissolved gradually as the reaction proceeded. Aliquots (*ca* 100 µL) were taken at 0, 1, 2, 3, 4, 5, 6, and 18 h; heated to 95 °C for several minutes; cooled down; and stored at −20 °C until HPLC analysis (Figure S3, Supplementary Material).

For the preparatory scale, 2,3-dehydrosilybin (equimolar mixture of enantiomers A and B, 200 mg, 0.417 mmol) was sulfated using the same procedure as for silybin for at least 6 h. Then, 2,3-dehydrosilybin-20-*O*-sulfate was obtained as a yellowish solid (22 mg, 9.4% yield, purity 95% Figure S5, RT 6.418 min, UV 254/368). The structure was determined by HRMS (*m*/*z* calcd. for C_25_H_19_O_13_S 559.05518, found 559.05454, Figure S4, Supplementary Material) and NMR (Table S1, Supplementary Material). Complete assignment of all NMR signals was achieved using COSY, HSQC, and HMBC experiments. The structure of 2,3-dehydrosilybin-7,20-*O*-disulfate (31 mg, 11.6% yield, purity 99%, Figure S7, RT 3.840 min, UV 255/371) was determined by HRMS (*m*/*z* calcd. for C_25_H_19_O_16_S_2_ 639.01200, found 639.01115, Figure S6, Supplementary Material) and ^13^C and ^1^H NMR (Table S2, Supplementary Material).

#### 3.4.3. Silychristin

Silychristin (200 mg, 0.415 mmol) was sulfated using the same procedure as for silybin. Silychristin-19-*O*-sulfate was obtained as a yellowish solid (75 mg, 32.3% yield, and purity 99.5%, Figure S10, Supplementary Material, RT 5.422 min, UV 208/288/335 nm). Its structure was determined by HRMS (*m*/*z* calcd. for C_25_H_21_O_13_S 561.07083, found 561.07092, Figure S9, Supplementary Material) and NMR (Table S3, Supplementary Material). As a side product of silychristin sulfation, 2,3-dehydrosilychristin-19-*O*-sulfate was always obtained as a yellow brownish solid (2.1 mg, 0.9% yield, purity 96%, Figure S12, Supplementary material, RT 6.756 min, UV 256/373 nm). Its structure was determined by HRMS (*m*/*z* calcd. for C_25_H_20_O_13_S 559.05518, found 559.05526, Figure S11, Supplementary Material) and NMR (Table S4, Supplementary Material).

Alternatively, 2,3-dehydrosilychristin-19-*O*-sulfate was prepared by oxidation of silychristin-19-*O*-sulfate using a modification of our optimized method for the preparation of 2,3-dehydrosilychristin [6]. Briefly, silychristin-19-*O*-sulfate (75 mg, 0.134 mmol) was dissolved in MeOH/H_2_O (4/1, 5 mL), 4-dimethylaminopyridine (DMAP, 15 mg, 0.123 mmol, 0.9 eq.) was added, and the mixture was stirred at 60 °C under an O_2_ atmosphere overnight. Then, 2,3-dehydrosilychristin-19-*O*-sulfate was obtained in 55% yield (41 mg, 0.73 mmol) and its identity was determined using HPLC profile, UV, MS, and NMR spectra.

A kinetic study of the time course of silychristin sulfation was performed according to the procedure used for 2,3-dehydrosilybin. Three reaction mixtures were prepared: (*i*) under Ar atmosphere (standard procedure), (*ii*) at low oxygen environment (3 × repeated freezing in liquid N_2_ under Ar atmosphere followed by degassing under high vacuum), and (*iii*) in 100% O_2_ atmosphere. All reaction mixtures were incubated at 30 °C in a rotary shaker and aliquots for HPLC analysis were taken at 0, 1, 2, 3, 4, 5, 6, and 21 h (Figure S8, Supplementary Material). Relative content of silychristin, silychristin-19-*O*-sulfate and 2,3-dehydrosilychristin-19-*O*-sulfate was determined from the AUC% from the respective HPLC chromatograms using the calibration curves of silychristin (for silychristin and silychristin-19-*O*-sulfate) and 2,3-dehydrosilychristin (for 2,3-dehydrosilychristin-19-*O*-sulfate).

#### 3.4.4. Silydianin

Silydianin (150 mg, 0.311 mmol) was sulfated using the same procedure as for silybin. Silydianin-19-*O*-sulfate was obtained as a yellowish solid (101 mg, 58.3%). Its structure was determined by HRMS (*m*/*z* calcd. for C_25_H_21_O_13_ S 561.07083, found 561.07141) and NMR (see Figure S13 and Table S5, Supplementary Material). This compound proved to be unstable and was not used for further biological activity testing.

### 3.5. Radical Scavenging and Anti-Lipoperoxidant Activities

*N*,*N*-dimethyl-*p*-phenylenediamine (DMPD) [33] and 2,2′-azino-*bis*(3-ethylbenzothiazolin-6-sulfonic acid (ABTS) [34] cation radical scavenging and ferric reducing antioxidant power (FRAP) [35] were measured using kits from Bioquochem (Llanera-Asturias, Spain). Antiradical activity by 2,2-diphenyl-1-picrylhydrazyl [36] radical scavenging assay, reducing capacity using Folin–Ciocalteau reagent (FCR) [37], and anti-lipoperoxidant activity using inhibition of lipid peroxidation of rat liver microsomes induced by *tert*-butyl hydroperoxide [38] were determined as described previously with modifications described in detail in our previous research [5,23,39,40,41].

### 3.6. Determination of Log p Values

The hydrophobicity of the compounds was determined by measuring their partition coefficient *P* in a mixture of two immiscible phases—octan-1-ol and 6.6 mM phosphate buffer pH 7.4. Stock solutions (0.5 mM) were prepared in octan-1-ol (parent compounds) and in the buffer (sulfated compounds). The stock solutions (150 µL) were mixed with 150 µL of the immiscible phase in microcentrifuge tubes (1.5 mL); shaken for 2 h (750 rpm, 25 °C); and after incubation, the phases were separated. Water phases were diluted 1:1 with the mobile phase (acetonitrile/deionized water/HCO_2_H, 5:95:0.1; *v*/*v*) and analyzed by HPLC using the gradient G3 (see Section 3.1.3). Octan-1-ol phases were evaporated in the stream of air (70 °C), then dissolved in 10 µL of DMSO, and mixed with 90 µL of mobile phase and analyzed by HPLC. The log *p* values were calculated from the respective peak heights (h) using the formula log *p* = log(h_octanol_/h_buffer_). For comparison, log *p* values were calculated using the Molinspiration property engine v2016.10 (http://www.molinspiration.com, Molinspiration Cheminformatics, Slovensky Grob, Slovakia) (access on 26th October 2017) [42]. 

### 3.7. Determination of NAD(P)H/Quinone Oxidoreductase 1 (NQO1) Activity in Hepa1c1c7 Cells

The murine hepatoma Hepa1c1c7 cell line (ECACC, Salisbury, UK) was cultured in minimum essential medium α (M0894, Sigma-Aldrich, St. Louis, MO, USA), supplemented with 2.2 g/L NaHCO_3_ and 10% heat- and charcoal-treated fetal bovine serum (Biochrom, Berlin, Germany), and maintained at 37 °C in a humidified atmosphere containing 5% CO_2_. For all experiments, cells were seeded onto 96-well plates at 1 × 10^4^ cells/well. After 24 h of stabilization, cells were treated for 48 h with 0.1% (*v*/*v*) DMSO (negative control), 2.5 µM sulforaphane (positive control; Sigma-Aldrich, St. Louis, MO, USA), or with the tested compounds in fresh culture medium. After the treatment, the activity of NQO1 was determined spectrophotometrically as described previously [43]. Cells were washed with phosphate-buffered saline (PBS) and lysed with 75 µL of digitonin solution (0.8 g/L digitonin, 2 mM EDTA, pH 7.8) by shaking on an orbital shaker for 20 min at room temperature. A part of the cell lysate (20 µL) was used to determine the protein content. The remaining lysate (55 µL) was mixed with 200 µL of 0.5 M Tris-Cl buffer containing 10% (*w*/*v*) bovine serum albumin, 1.5% (*v*/*v*) Tween-20, 7.5 mM FAD, 150 mM glucose-6-phosphate, 2 U/mL glucose-6-phosphate dehydrogenase (Roche Diagnostics, Mannheim, Germany), 50 mM NADP^+^, 25 mM menadione, and 0.7 mM 3-(4,5-dimethylthiazol-2-yl)-2,5-diphenyltetrazolium bromide (MTT). The mixture was incubated for 5 min at room temperature and the reaction was stopped with 50 µL of dicumarol suspension (0.3 mM dicumarol, 5 mM potassium phosphate, 0.5% (*v*/*v*) DMSO). The absorbance of the reduced MTT corresponding to the activity of NQO1 was measured at 610 nm on a spectrophotometric plate reader. The absorbance values were normalized to the protein content and used for the calculation of fold changes versus the control.

The possible cytotoxicity of the tested compounds was checked using the MTT reduction assay. In brief, Hepa1c1c7 cells were seeded and treated as described above. Positive controls were treated for 48 h with 1.5% (*v*/*v*) Triton X-100. After the treatment, cells were washed with PBS and incubated for 2 h at 37 °C in serum-free medium containing 0.5 mg/mL MTT. After incubation, the medium was removed and formazan produced by active mitochondria was dissolved in the mixture of DMSO and 25% (*m*/*m*) NH_4_OH (99:1; *v*/*v*). The absorbance was measured at 540 nm and used to calculate relative cell viability, where cells treated with 0.1% (*v*/*v*) DMSO (negative control) represented 100% viability.

## 4. Statistical Analysis

All data were analyzed with one-way ANOVA, Scheffé and least square difference tests for post hoc comparisons among pairs of means using the statistical package Statext ver. 2.1 (STATEXT LLC, Wayne, NJ, USA). Differences were considered statistically significant when *p* < 0.05. 

## 5. Conclusions

In the present work, we succeeded in the preparation of sulfated metabolites of silymarin flavonolignans, namely silybin A 20-*O*-sulfate, silybin B 20-*O*-sulfate, 2,3-dehydrosilybin-20-*O*-sulfate, 2,3-dehydrosilybin-7,20-di-*O*-sulfate, silychristin-19-*O*-sulfate, and 2,3-dehydrosilychristin-19-*O*-sulfate (silydianin-19-*O*-sulfate was unstable). As expected, the sulfation had a mostly diminishing effect on the radical scavenging and anti-lipoperoxidant activity of the flavonolignans. However, most sulfates retained some redox and antiradical activity and in some assays, such as FRAP and DMPD radical scavenging, 2,3-dehydrosilybin-20-*O*-sulfate, 2,3-dehydrosilybin-7,20-di-*O*-sulfate, and 2,3-dehydrosilychristin-19-*O*-sulfate were even more active than the parent compounds. The sulfated flavonolignans obtained will be employed for deeper investigation of their biological activity and as authentic standards for metabolic studies with silymarin. Silybin A-20-*O*-sulfate, silybin B-20-*O*-sulfate, 2,3-dehydrosilybin-20-*O*-sulfate, and silychristin-19-*O*-sulfate were recently proven to be formed by isolated human hepatocytes incubated with respective silymarin constituents [44]; therefore, they can be considered to be authentic human metabolites. This study should contribute to better understanding of the metabolic transformations of major components of silymarin that are broadly used as a food supplement and nutraceutical.

## Figures and Tables

**Table 1:** Overview of aryl sulfotransferase from rat liver (AST IV) substrate specificity and isolated sulfated products.

Substance	Formation of *p*-NP	Isolated Product(s)
Silybin A/B	+ ^a^	Silybin B 20-*O*-sulfate ^b^
Silybin A	– ^c^	− ^c^
Silybin B	+ ^a^	Silybin B 20-*O*-sulfate
Silychristin	+ ^a^	– ^c^
Silydianin	+ ^a^	– ^c^

^a^ isolated sulfated product(s) and/or release of *p*-nitrophenol (*p*-NP), ^b^ the case of natural silybin, only silybin B 20-*O*-sulfate was isolated, ^c^ no formation of *p*-NP and/or isolated sulfated products.

**Table 2:** Isolated flavonolignan sulfates, their purity, analytical characteristics, and yields.

Compound	Purity [%] *	λ_max_ [nm] ^†^	HRMS-ESI *m*/*z*	RT [min] *	Isolated Yield	
					[mg/ 100 mg]	[mol%]
Silybin A-20-*O*-sulfate	99	204/285	561.07037	3.835	65.5	56
Silybin B 20-O-sulfate	99.9	203/285	561.07068	3.840	46.5	40
2,3-Dehydrosilybin-20-*O*-sulfate	95	254/368	559.05454	6.418	11	10
2,3-Dehydrosilybin-7,20-*O*-disulfate	99	255/371	639.01115	3.439	15.5	12
Silychristin-19-*O*-sulfate	99.5	208/288/335	561.07092	5.422	37.5	32
2,3-Dehydrosilychristin-19-*O*-sulfate	96	256/373	559.05526	6.756	1.1 (54) ^$^	0.9.(55) ^$^
Silydianin-19-*O*-sulfate	ND ^‡^	ND ^‡^	ND ^‡^	ND ^‡^	55	58

* by HPLC, ^†^ by photodiode array (PDA) detector of HPLC, ^$^ by oxidation of silychristin-19-*O*-sulfate, ^‡^ not determined (ND) because of the instability of the compound.

**Table 3:** Radical scavenging and reducing capacity of flavonolignan sulfates in comparison with non-conjugated flavonolignans.

Compound	DPPH ^a^ IC_50_ [μM]	ABTS^+ b^ [CE]	FCR ^c^ [GAE]	DMPD^+ d^ [CE]	FRAP ^e^ [Fe^2+^]
Silybin A	490 ± 21 ^f^	1.01 ± 0.03 ^i^	0.33 ± 0.02 ^k^	0.96 ± 0.01 ^n^	0.06 ± 0.00
Silybin A 20-*O*-sulfate	>2500	0.02 ± 0.00	0.08 ± 0.01	0.98 ± 0.01 ^n^	0.01 ± 0.00 ^p^
Silybin B	546 ± 17 ^f^	0.97 ± 0.05 ^i^	0.36 ± 0.03 ^k^	1.09 ± 0.01 ^n^	0.04 ± 0.00 ^q^
Silybin B 20-*O*-sulfate	>2500	0.04 ± 0.00	0.16 ± 0.02	0.99 ± 0.01 ^n^	0.01 ± 0.00 ^p^
2,3-Dehydrosilybin	13.3 ± 0.6 ^g^	0.77 ± 0.01 ^j^	1.51 ± 0.09 ^l^	1.02 ± 0.02 ^n^	0.81 ± 0.02
2,3-Dehydrosilybin-20-*O*-sulfate	14.1 ± 0.5 ^g^	0.71 ± 0.03 ^j^	1.03 ± 0.01 ^m^	0.97 ± 0.02 ^n^	1.46 ± 0.03 ^r^
2,3-Dehydrosilybin-7,20-di-*O*-sulfate	106 ± 4	0.55 ± 0.03	0.95 ± 0.01 ^m^	1.90 ± 0.10	1.61 ± 0.04
Silychristin	37.1 ± 3.1	1.50 ± 0.09	1.13 ± 0.23 ^m^	1.50 ± 0.02^o^	0.64 ± 0.06
Silychristin-19-*O*-sulfate	587 ± 11 ^f^	0.62 ± 0.03 ^k^	0.80 ± 0.11 ^m^	1.56 ± 0.08^o^	0.05 ± 0.01 ^q^
2,3-Dehydrosilychristin	8.6 ± 0.8 ^h^	0.93 ± 0.03 ^l^	1.58 ± 0.04 ^l^	0.97 ± 0.02 ^n^	0.28 ± 0.01
2,3-Dehydro-silychristin-19-*O*-sulfate	7.9 ± 0.3 ^h^	0.62 ± 0.02 ^n^	1.44 ± 0.01 ^l^	1.02 ± 0.04 ^n^	1.44 ± 0.04 ^r^
Trolox	2.9 ± 0.1	ND	0.33 ± 0.00 ^k^	ND	ND

Data are presented as mean ± standard error from at least three independent measurements performed in triplicate. ^a^ 1,1-Diphenyl-2-picrylhydrazyl, ^b^ 2,2′-azino-*bis*-(3-ethylbenzothiazoline-6-sulfonic acid) cation radical scavenging (vitamin C equivalents), ^c^ Folin–Ciocalteau reagent reduction (gallic acid equivalents), ^d^ *N*,*N*-dimethyl-*p*-phenylenediamine cation radical scavenging (vitamin C equivalents), ^e^ ferric reducing antioxidant power, ^f–r^ The values marked with the same letter are not significantly different. ND = not determined.

**Table 4:** Anti-lipoperoxidant capacity and lipophilicity of flavonolignan sulfates in comparison with non-conjugated flavonolignans.

Compound	Lpx ^a^ IC_50_ [μM]	Log *p*	
		Exp ^b^	Pred ^c^
Silybin A	48.6 ± 0.4	1.52	1.47
Silybin A 20-*O*-sulfate	>1000	−1.65	−2.03
Silybin B	68.6 ± 2.2	2.17	1.47
Silybin B 20-*O*-sulfate	>1000	−2.34	−2.03
2,3-Dehydrosilybin	10.4 ± 0.4	>3 ^e^	2.44
2,3-Dehydrosilybin-20-*O*-sulfate	13.7 ± 0.4 ^d^	−2.16	−1.06
2,3-Dehydrosilybin-7,20-di-*O*-sulfate	90.7 ± 3.3	<−3 ^f^	−1.65
Silychristin	17.9 ± 0.7	1.47	1.26
Silychristin-19-*O*-sulfate	134 ± 2	−2.25	−2.23
2,3-Dehydrosilychristin	14.6 ± 0.4 ^d^	>3 ^e^	2.24
2,3-Dehydro-silychristin-19-*O*-sulfate	12.0 ± 1.8 ^d^	−2.24	−1.26
Trolox	32.7 ± 2.4	ND	1.63

Data are presented as mean ± standard error from at least three independent measurements performed in triplicate. ^a^ Inhibition of lipoperoxidation (Lpx) of rat liver microsomal membranes induced by *tert*-butyl hydroperoxide. ^b^ Experimental, ^c^ predicted using the Molinspiration property engine. ^d^ The values are not significantly different. ^e,f^ Concentration of the compound in the aqueous or octanol phase, respectively, was below the quantification limit. ND = not determined.

## Supplementary Materials

Supplementary materials can be found at http://www.mdpi.com/1422-0067/19/8/2349/s1.

## Abbreviations

ABTS	2,2′-azinobis-(3-ethylbenzothiazoline-6-sulfonic acid)
AST	aryl sulfotransferase
AST DH	aryl sulfotransferase from *Desulfitobacterium hafniense*
AST IV	aryl sulfotransferase from rat liver
CE	vitamin C equivalents
COSY	correlation spectroscopy
DPPH	1,1-diphenyl-2-picrylhydrazyl
DMPD	*N*,*N*-dimethyl-*p*-phenylenediamine
DMAP	4-dimethylaminopyridine
DMSO	dimethylsulfoxide
FCR	Folin–Ciocalteu reagent
FRAP	ferric reducing antioxidant power
GAE	gallic acid equivalents
HMBC	heteronuclear multiple-bond correlation spectroscopy
HSQC	heteronuclear single-quantum correlation spectroscopy
IC_50_	the concentration of the tested compound that inhibited the reaction by 50%
Lpx	lipid peroxidation
MTT	3-(4,5-dimethylthiazol-2-yl)-2,5-diphenyltetrazolium bromide
NQO1	NAD(P)H/quinone oxidoreductase 1
Nrf2	nuclear factor erythroid 2-related factor 2
PBS	phosphate-buffered saline
*p*-NP	*p*-nitrophenol
*p*-NPS	*p*-nitrophenyl sulfate
PDA	photodiode array

## References

[1] Abenavoli L., Capasso R., Milic N., Capasso F. (2010). Milk thistle in liver diseases: Past, present, future. Phytother. Res..

[2] Gažák R., Walterová D., Křen V. (2007). Silybin and silymarin-new and emerging applications in medicine. Curr. Med. Chem..

[3] Biedermann D., Vavříková E., Cvak L., Křen V. (2014). Chemistry of silybin. Nat. Prod. Rep..

[4] Wagner H., Seligmann O., Seitz M., Abraham D., Sonnenbichler J. (1976). Silydianin and silychristin, 2 isomeric silymarins from *Silybum marianum* L. Gaertn (milk thistle). Z. Naturforsch. B.

[5] Pyszková M., Biler M., Biedermann D., Valentová K., Kuzma M., Vrba J., Ulrichová J., Sokolová R., Mojovic M., Popovic-Bijelic A. (2016). Flavonolignan 2,3-dehydroderivatives: Preparation, antiradical and cytoprotective activity. Free Radic. Biol. Med..

[6] Biedermann D., Buchta M., Holečková V., Sedlák D., Valentová K., Cvačka J., Bednárová L., Křenková A., Kuzma M., Škuta C. (2016). Silychristin: Skeletal alterations and biological activities. J. Nat. Prod..

[7] Křenek K., Marhol P., Peikerová Ž., Křen V., Biedermann D. (2014). Preparatory separation of the silymarin flavonolignans by Sephadex LH-20 gel. Food Res. Int..

[8] Egea J., Fabregat I., Frapart Y.M., Ghezzi P., Görlach A., Kietzmann T., Kubaichuk K., Knaus U.G., Lopez M.G., Olaso-Gonzalez G. (2017). European contribution to the study of ROS: A summary of the findings and prospects for the future from the COST action BM1203 (EU-ROS). Redox Biol..

[9] Moosavi F., Hosseini R., Saso L., Firuzi O. (2016). Modulation of neurotrophic signaling pathways by polyphenols. Drug Des. Dev. Ther..

[10] Křen V., Marhol P., Purchartová K., Gabrielová E., Modrianský M. (2013). Biotransformation of silybin and its congeners. Curr. Drug Metab..

[11] Theodosiou E., Purchartová K., Stamatis H., Kolisis F., Křen V. (2014). Bioavailability of silymarin flavonolignans: Drug formulations and biotransformation. Phytochem. Rev..

[12] Miranda S.R., Lee J.K., Brouwer K.L.R., Wen Z.M., Smith P.C., Hawke R.L. (2008). Hepatic metabolism and biliary excretion of silymarin flavonolignans in isolated perfused rat livers: Role of multidrug resistance-associated protein 2 (abcc2). Drug Metab. Dispos..

[13] Kr̆en V., Ulrichová J., Kosina P., Stevenson D., Sedmera P., Přikrylová V., Halada P., Šimánek V. (2000). Chemoenzymatic preparation of silybin β-glucuronides and their biological evaluation. Drug Metab. Dispos..

[14] Marhol P., Bednář P., Kolářová P., Večeřa R., Ulrichová J., Tesařová E., Vavříková E., Kuzma M., Křen V. (2015). Pharmacokinetics of pure silybin diastereoisomers and identification of their metabolites in rat plasma. J. Funct. Food..

[15] Almeida A.F., Borge G.I.A., Piskula M., Tudose A., Tudoreanu L., Valentová K., Williamson G., Santos C.N. (2018). Bioavailability of quercetin in humans with a focus on interindividual variation. Compr. Rev. Food Sci. Food Saf..

[16] Gonzalez-Sarrias A., Garcia-Villalba R., Romo-Vaquero M., Alasalvar C., Orem A., Zafrilla P., Tomas-Barberan F.A., Selma M.V., Espin J.C. (2017). Clustering according to urolithin metabotype explains the interindividual variability in the improvement of cardiovascular risk biomarkers in overweight-obese individuals consuming pomegranate: A randomized clinical trial. Mol. Nutr. Food Res..

[17] Marhol P., Hartog A.F., van der Horst M.A., Wever R., Purchartová K., Fuksová K., Kuzma M., Cvačka J., Křen V. (2013). Preparation of silybin and isosilybin sulfates by sulfotransferase from *Desulfitobacterium hafniense*. J. Mol. Catal. B-Enzym..

[18] Purchartová K., Engels L., Marhol P., Šulc M., Kuzma M., Slámová K., Elling L., Křen V. (2013). Enzymatic preparation of silybin phase II metabolites: Sulfation using aryl sulfotransferase from rat liver. Appl. Microbiol. Biot..

[19] Purchartová K., Valentová K., Pelantová H., Marhol P., Cvačka J., Havlíček L., Křenkova A., Vavříková E., Biedermann D., Chambers C.S. (2015). Prokaryotic and eukaryotic aryl sulfotransferases: Sulfation of quercetin and its derivatives. ChemCatChem.

[20] Trouillas P., Marsal P., Svobodová A., Vostálová J., Gažák R., Hrbáč J., Sedmera P., Křen V., Lazzaroni R., Duroux J.-L. (2008). Mechanism of the antioxidant action of silybin and 2,3-dehydrosilybin flavonolignans: A joint experimental and theoretical study. J. Phys. Chem. A.

[21] Gažák R., Trouillas P., Biedermann D., Fuksová K., Marhol P., Kuzma M., Křen V. (2013). Base-catalyzed oxidation of silybin and isosilybin into 2,3-dehydro derivatives. Tetrahedron Lett..

[22] Kawabata K., Mukai R., Ishisaka A. (2015). Quercetin and related polyphenols: New insights and implications for their bioactivity and bioavailability. Food Funct..

[23] Valentová K., Káňová K., Di Meo F., Pelantová H., Chambers C., Rydlová L., Petrásková L., Křenková A., Cvačka J., Trouillas P. (2017). Chemoenzymatic preparation and biophysical properties of sulfated quercetin metabolites. Int. J. Mol. Sci..

[24] Roubalová L., Purchartová K., Papoušková B., Vacek J., Křen V., Ulrichová J., Vrba J. (2015). Sulfation modulates the cell uptake, antiradical activity and biological effects of flavonoids in vitro: An examination of quercetin, isoquercitrin and taxifolin. Bioorg. Med. Chem..

[25] Seyoum A., Asres K., El-Fiky F.K. (2006). Structure–radical scavenging activity relationships of flavonoids. Phytochemistry.

[26] Chambers C.S., Holečková V., Petrásková L., Biedermann D., Valentová K., Buchta M., Křen V. (2017). The silymarin composition… and why does it matter???. Food Res. Int..

[27] Surh Y.J., Kundu J.K., Na H.K. (2008). Nrf2 as a master redox switch in turning on the cellular signaling involved in the induction of cytoprotective genes by some chemopreventive phytochemicals. Planta Med..

[28] Zhang Y., Talalay P., Cho C.G., Posner G.H. (1992). A major inducer of anticarcinogenic protective enzymes from broccoli: Isolation and elucidation of structure. Proc. Natl. Acad. Sci. USA.

[29] Roubalová L., Dinkova-Kostova A.T., Biedermann D., Křen V., Ulrichová J., Vrba J. (2017). Flavonolignan 2,3-dehydrosilydianin activates Nrf2 and upregulates NAD(P)H:quinone oxidoreductase 1 in Hepa1c1c7 cells. Fitoterapia.

[30] Gažák R., Marhol P., Purchartová K., Monti D., Biedermann D., Riva S., Cvak L., Křen V. (2010). Large-scale separation of silybin diastereoisomers using lipases. Process Biochem..

[31] Džubák P., Hajdúch M., Gažák R., Svobodová A., Psotová J., Walterová D., Sedmera P., Křen V. (2006). New derivatives of silybin and 2,3-dehydrosilybin and their cytotoxic and P-glycoprotein modulatory activity. Bioorg. Med. Chem..

[32] Van der Horst M.A., van Lieshout J.F.T., Bury A., Hartog A.F., Wever R. (2012). Sulfation of various alcoholic groups by an arylsulfate sulfotransferase from *Desulfitobacterium hafniense* and synthesis of estradiol sulfate. Adv. Synth. Catal..

[33] Fogliano V., Verde V., Randazzo G., Ritieni A. (1999). Method for measuring antioxidant activity and its application to monitoring the antioxidant capacity of wines. J. Agric. Food Chem..

[34] Miller N.J., RiceEvans C.A. (1997). Factors influencing the antioxidant activity determined by the ABTS+ radical cation assay. Free Radic. Res..

[35] Jones A., Pravadali-Cekic S., Dennis G.R., Bashir R., Mahon P.J., Shalliker R.A. (2017). Ferric reducing antioxidant potential (FRAP) of antioxidants using reaction flow chromatography. Anal. Chim. Acta.

[36] Sharma O.P., Bhat T.K. (2009). DPPH antioxidant assay revisited. Food Chem..

[37] Singleton V.L., Orthofer R., Lamuela-Raventós R.M. (1999). Analysis of total phenols and other oxidation substrates and antioxidants by means of folin-ciocalteu reagent. Methods in Enzymol.

[38] Joyeux M., Lobstein A., Anton R., Mortier F. (1995). Comparative antilipoperoxidant, antinecrotic and scavenging properties of terpenes and biflavones from Ginkgo and some flavonoids. Planta Med..

[39] Heřmánková-Vavříková E., Křenková A., Petrásková L., Chambers C., Zápal J., Kuzma M., Valentová K., Křen V. (2017). Synthesis and antiradical activity of isoquercitrin esters with aromatic acids and their homologues. Int. J. Mol. Sci..

[40] Vavříková E., Křen V., Jezova-Kalachova L., Biler M., Chantemargue B., Pyszková M., Riva S., Kuzma M., Valentová K., Ulrichová J. (2017). Novel flavonolignan hybrid antioxidants: From enzymatic preparation to molecular rationalization. Eur. J. Med. Chem..

[41] Vavříková E., Langschwager F., Jezova-Kalachova L., Křenková A., Mikulová B., Kuzma M., Křen V., Valentová K. (2016). Isoquercitrin esters with mono- or dicarboxylic acids: Enzymatic preparation and properties. Int. J. Mol. Sci..

[42] Ertl P., Rohde B., Selzer P. (2000). Fast calculation of molecular polar surface area as a sum of fragment-based contributions and its application to the prediction of drug transport properties. J. Med. Chem..

[43] Fahey J.W., Dinkova-Kostova A.T., Stephenson K.K., Talalay P. (2004). The “Prochaska” microtiter plate bioassay for inducers of NQO1. Methods in Enzymol.

[44] Vrba J., Papoušková B., Roubalová L., Zatloukalová M., Biedermann D., Křen V., Valentová K., Ulrichová J., Vacek J. (2018). Metabolism of flavonolignans in human hepatocytes. J. Pharm. Biomed. Anal..

