# Supplementary material for: Sulfated Metabolites of Flavonolignans and 2,3-Dehydroflavonolignans: Preparation and Properties
Source: Int J Mol Sci. 2018 Aug 9;19(8):2349. doi: 10.3390/ijms19082349 (PMC6121260; doi:10.3390/ijms19082349)
Supplement: Supplementary file 1 [file ijms-19-02349-s001.pdf]

Sulfated Metabolites of Flavonolignans and 2,3-Dehydroflavonolignans: Preparation and Properties

Kateřina Valentová [1,*] , Kateřina Purchartová [1,2], Lenka Rydlová [1,2], Lenka Roubalová [3,4], David Biedermann [1], Lucie Petrásková [1], Alena Křenková [1], Helena Pelantová [1], Veronika Holečková-Moravcová [1], Eva Tesařová [2], Josef Cvačka [5], Jiří Vrba [3,4], Jitka Ulrichová [3,4], Vladimír Křen [1]

[1] Institute of Microbiology of the Czech Academy of Sciences, Vídeňská 1083, 14220 Prague, Czech Republic; katerina.purchartova@gmail.com (K.P.), rydlova.l@email.cz (L.Ry.), biedermann@biomed.cas.cz (D.B.), petraskova@biomed.cas.cz (L.P.), alenka.petrickova@gmail.com (A.K.), pelantova@biomed.cas.cz (H.P.), vholeckov@gmail.com (V.H.-M.), kren@biomed.cas.cz (V.K.)

[2] Faculty of Science, Charles University, Department of Physical and Macromolecular Chemistry, Hlavova 2030/8, 12843 Prague, Czech Republic; eva.tesarova@natur.cuni.cz (E.T.)

[3] Department of Medical Chemistry and Biochemistry, Faculty of Medicine and Dentistry, Palacký University, Hněvotínská 3, Olomouc 77515, Czech Republic; roubalova.lenka@seznam.cz (L.Ro.), vrbambv@seznam.cz (J.V.), jitkaulrichova@seznam.cz (J.U.)

[4] Institute of Molecular and Translational Medicine, Faculty of Medicine and Dentistry, Palacký University, Hněvotínská 3, Olomouc 77515, Czech Republic

[5] Institute of Organic Chemistry and Biochemistry of the Czech Academy of Sciences, Flemingovo nám. 2, 16610 Prague, Czech Republic; josef.cvacka@uochb.cas.cz

* Correspondence: kata.valentova@email.cz; Tel: +420-296-442-509.

Table of Contents

Figure S1. HPLC chromatogram of silybin A 20-*O*-sulfate ... 3

Figure S2. HPLC chromatogram of silybin B 20-*O*-sulfate ... 3

Figure S3. HPLC chromatograms of 2,3-dehydrosilybin A sulfation in time ... 4

Figure S4. ESI-MS− spectrum of 2,3-dehydrosilybin 20-*O*-sulfate ... 5

Table S1. ^{13}C and ^{1}H NMR data of 2,3-dehydrosilybin-20-*O*-sulfate ... 5

Figure S5. HPLC chromatogram of 2,3-dehydrosilybin-20-*O*-sulfate ... 6

Figure S6. ESI-MS− spectrum of 2,3-dehydrosilybin-7,20-di-*O*-sulfate ... 6

Table S2. ^{13}C and ^{1}H NMR data of 2,3-dehydrosilybin-7,20-di-*O*-sulfate ... 7

Figure S7. HPLC chromatogram of 2,3-dehydrosilybin-7,20-di-*O*-sulfate ... 7

Figure S8. HPLC chromatograms of silychristin sulfation after 1 h of reaction ... 8

Figure S9. ESI-MS− spectrum of silychristin-19-*O*-sulfate ... 8

Table S3. ^{13}C and ^{1}H NMR data of silychristin-19-*O*-sulfate ... 9

Figure S10. HPLC chromatogram of silychristin-19-*O*-sulfate ... 9

Figure S11. ESI-MS− spectrum of 2,3-dehydrosilychristin-19-*O*-sulfate ... 10

Table S4. ^{13}C and ^{1}H NMR data of 2,3-dehydrosilychristin-19-*O*-sulfate ... 10

Figure S12. HPLC chromatogram of 2,3-dehydrosilychristin-19-*O*-sulfate ... 11

Figure S13. ESI-MS− spectrum of silydianin 19-*O*-sulfate ($[M – H]^-$, *m/z* 561.1) ... 11

Table S5. ^{13}C and ^{1}H NMR data of silydianin 19-*O*-sulfate ... 12

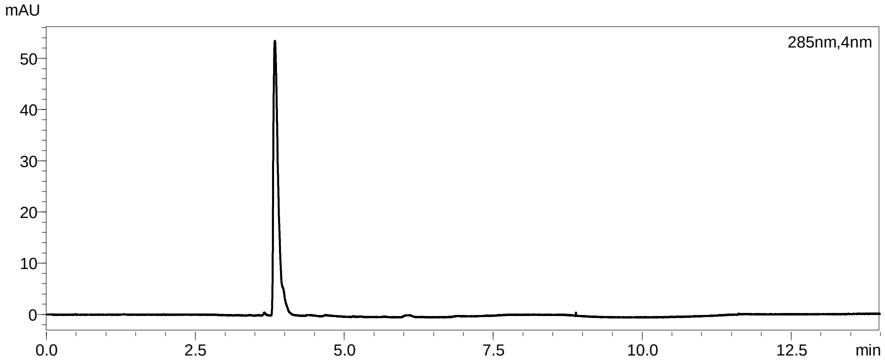

Figure S1. HPLC chromatogram of silybin A 20-*O*-sulfate.

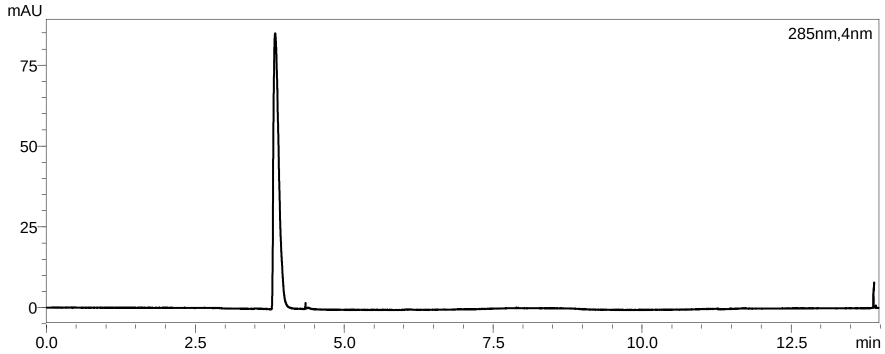

Figure S2. HPLC chromatogram of silybin B 20-*O*-sulfate.

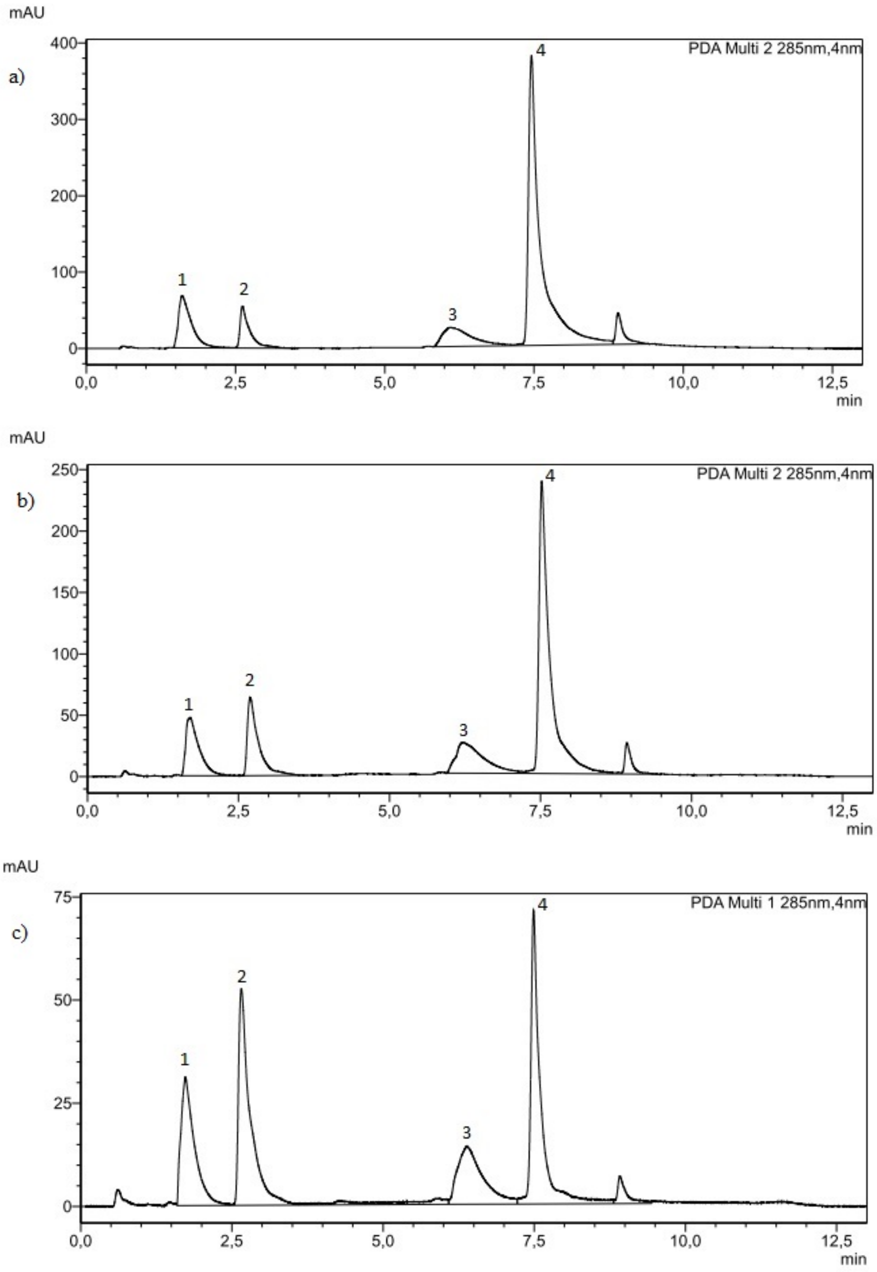

Figure S3. HPLC chromatograms of 2,3-dehydrosilybin A sulfation in time.
a) 1h, b) 5h, c) 12h; 1 – *p*-NPS, 2 – *p*-NP, 3 – 2,3-dehydrosilybin A sulfate, 4 – 2,3-dehydrosilybin

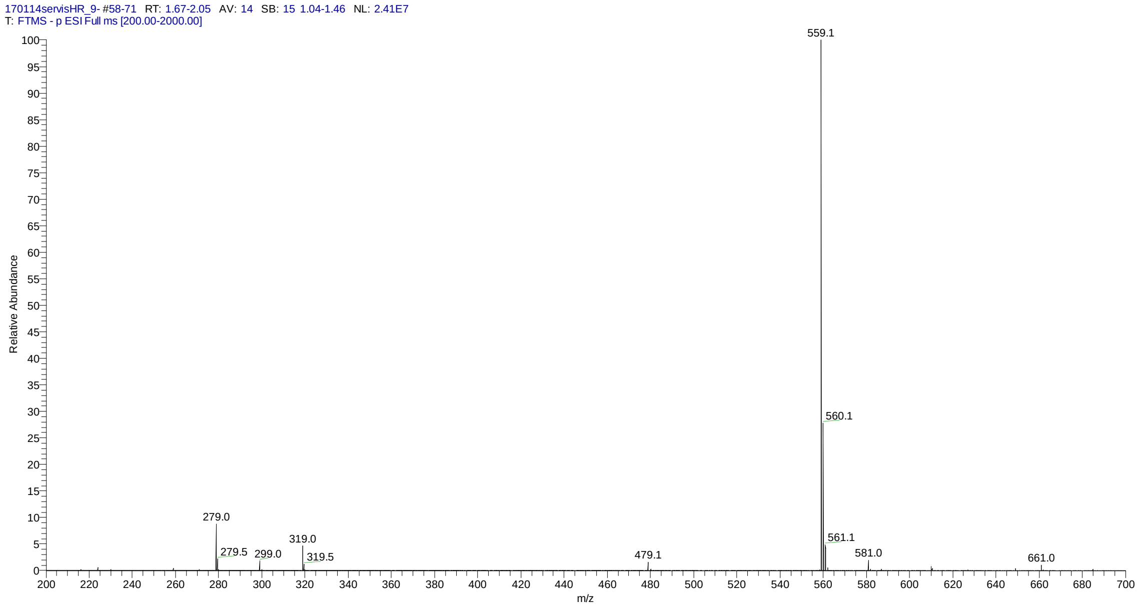

Figure S4. ESI-MS− spectrum of 2,3-dehydrosilybin 20-*O*-sulfate. ([M – H]$^-$, *m/z* 559.1; [M – 2H]^2, *m/z* 279.0)

Table S1. ^{13}C and ^{1}H NMR data of 2,3-dehydrosilybin-20-*O*-sulfate. (600.23 MHz for ^{1}H, 150.95 MHz for ^{13}C, DMSO-d_6, 30 °C)

Atom	δ_C	m.	δ_H	n(H)	m.	*J* [Hz]
**2**	145.83	S	-	0		
**3**	136.45	S	-	0		
**4**	176.11	S	-	0		
**4a**	103.14	S	-	0		
**5**	160.74	S	-	0		
**6**	98.33	D	6.186	1	d	2.0
**7**	164.14	S	-	0		
**8**	93.64	D	6.461	1	d	2.0
**8a**	156.29	S	-	0		
**10**	78.52	D	4.303	1	ddd	7.9, 4.6, 2.5
**11**	75.81	D	5.018	1	d	7.9
**12a**	143.36	S	-	0		
**13**	116.22	D	7.780	1	m	
**14**	123.87	S	-	0		
**15**	121.42	D	7.773	1	m	
**16**	116.92	D	7.127	1	m	
**16a**	145.06	S	-	0		
**17**	131.24	S	-	0		
**18**	112.06	D	7.084	1	d	2.0
**19**	150.44	S	-	0		
**20**	143.28	S	-	0		
**21**	120.59	D	7.513	1	d	8.4
**22**	119.67	D	6.968	1	dd	8.4, 2.0
**23**	60.08	T	3.594	1	ddd	12.4, 4.8, 2.5
			3.391	1	ddd	12.4, 5.8, 4.6
**3-OH**	-	-	9.530	1	br s	
**5-OH**	-	-	12.406	1	s	
**7-OH**	-	-	10.803	1	br s	
**19-OMe**	55.82	Q	3.769	3	s	
**23-OH**	-	-	5.010	1	dd	5.8, 4.8

m. multiplicity

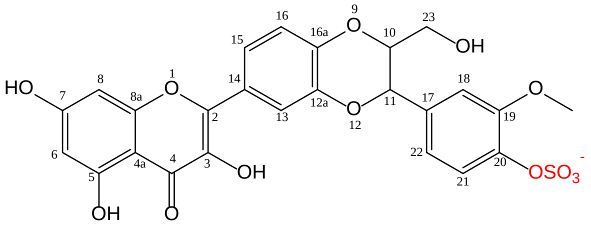

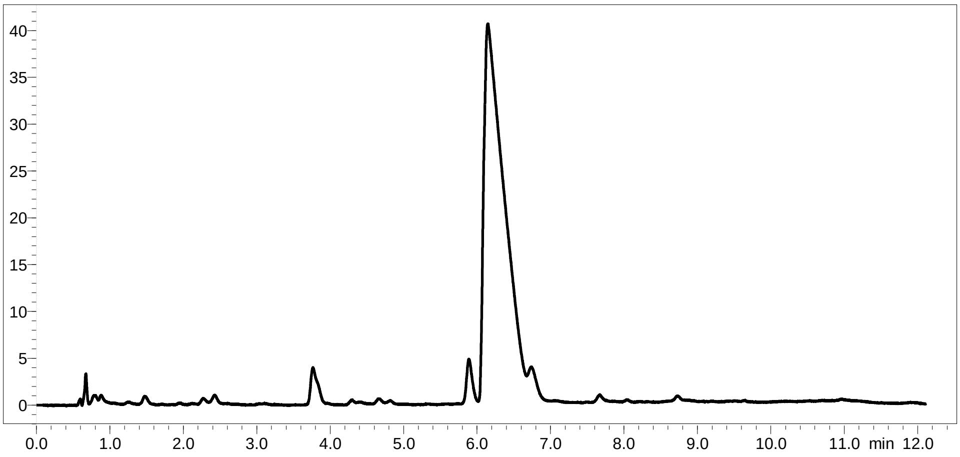

Figure S5. HPLC chromatogram of 2,3-dehydrosilybin-20-*O*-sulfate.

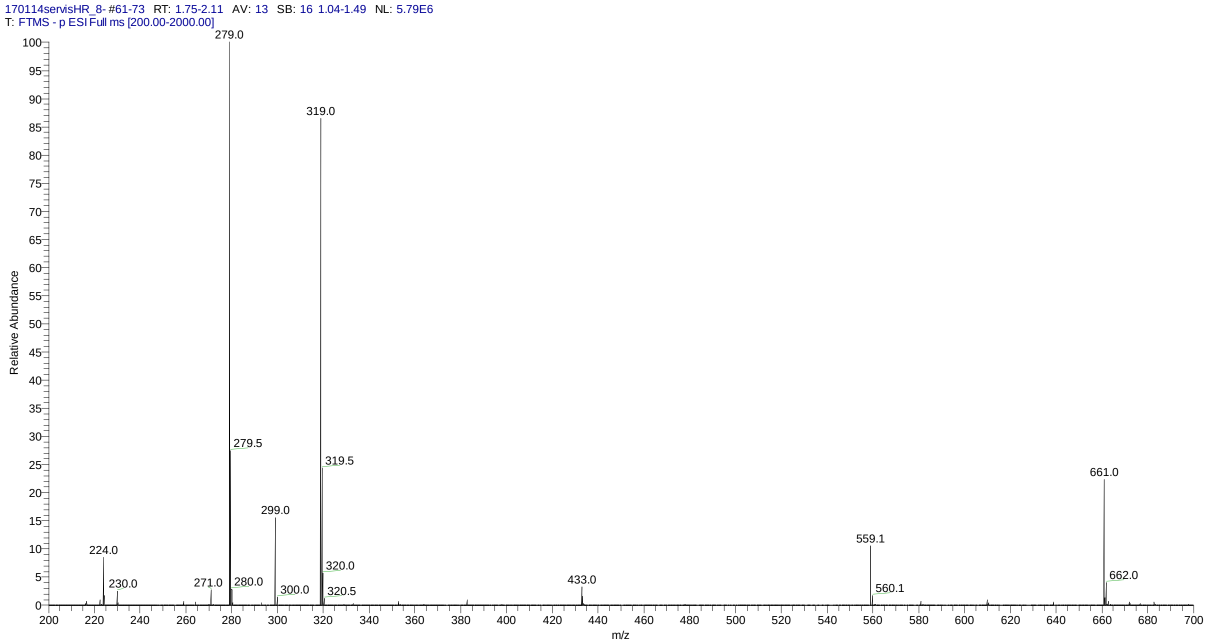

Figure S6. ESI-MS− spectrum of 2,3-dehydrosilybin-7,20-di-*O*-sulfate.
([M – 2H + Na] *m/z* 661.0; $[M-H-SO_3]^-$, *m/z* 559.1; $[M-2H]^{2-}$, *m/z* 319.0; $[M-2H-SO_3]^{2-}$, *m/z* 279.0)

Table S2. ^{13}C and ^{1}H NMR data of 2,3-dehydrosilybin-7,20-di-*O*-sulfate. (600.23 MHz for ^{1}H, 150.95 MHz for ^{13}C, DMSO-d_6, 30 °C)

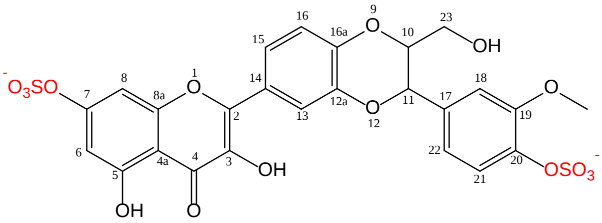

Atom	δ_C	m.	δ_H	n(H)	m.	*J* [Hz]
**2**	146.60	S	-	0		
**3**	136.78	S	-	0		
**4**	176.42	S	-	0		
**4a**	105.13	S	-	0		
**5**	159.80	S	-	0		
**6**	101.45	D	6.607	1	d	2.0
**7**	159.49	S	-	0		
**8**	97.70	D	6.958	1	d	2.0
**8a**	155.27	S	-	0		
**10**	78.56	D	4.298	1	ddd	7.9, 4.6, 2.5
**11**	75.79	D	5.026	1	d	7.9
**12a**	143.30	S	-	0		
**13**	116.68	D	7.869	1	m	
**14**	123.75	S	-	0		
**15**	121.25	D	7.757	1	m	
**16**	116.99	D	7.136	1	m	
**16a**	145.19	S	-	0		
**17**	131.22	S	-	0		
**18**	111.98	D	7.087	1	d	2.0
**19**	150.43	S	-	0		
**20**	143.24	S	-	0		
**21**	120.59	D	7.510	1	d	8.4
**22**	119.64	D	6.969	1	dd	8.4, 2.0
**23**	60.07	T	3.591	1	ddd	12.4, 5.0, 2.5
			3.385	1	ddd	12.4, 5.9, 4.6
**3-OH**	-	-	9.644[a]	1	br s	
**5-OH**	-	-	12.325	1	s	
**7-OH**	-	-	n.d.[a]	-		
**19-OMe**	55.80	Q	3.768	3	s	
**23-OH**	-	-	5.015	1	dd	5.9, 5.0

[a] - tentative assignment, m. - multiplicity

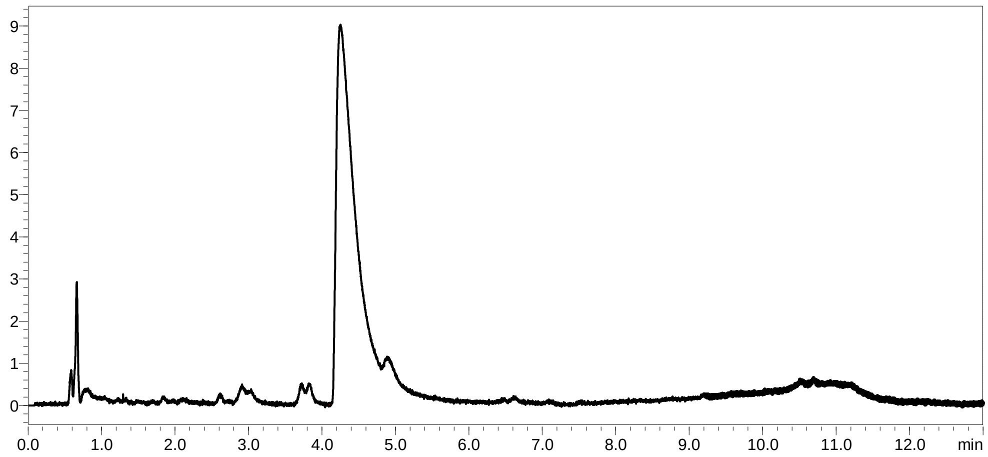

Figure S7. HPLC chromatogram of 2,3-dehydrosilybin-7,20-di-*O*-sulfate.

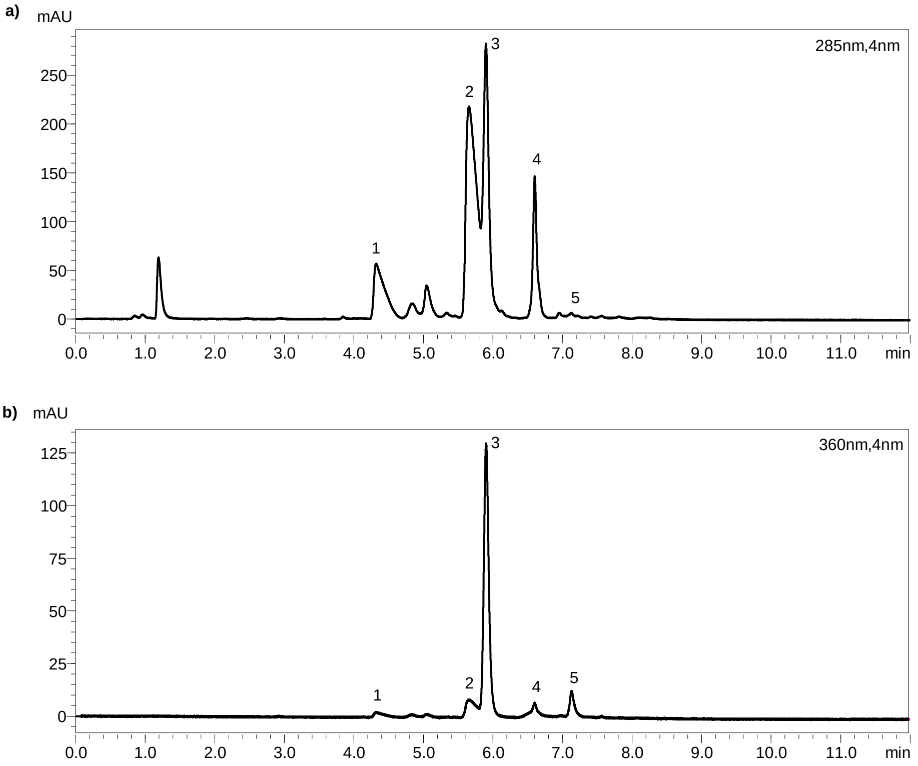

Figure S8. HPLC chromatograms of silychristin sulfation after 1 h of reaction under Ar atmosphere. Recorded at a) 285 nm, b) 360 nm; 1 – *p*-NPS, 2 – silychristin-19-*O*-sulfate, 3 – *p*-NP, 4 – silychristin, 5 – 2,3-dehydrosilychristin-19-*O*-sulfate

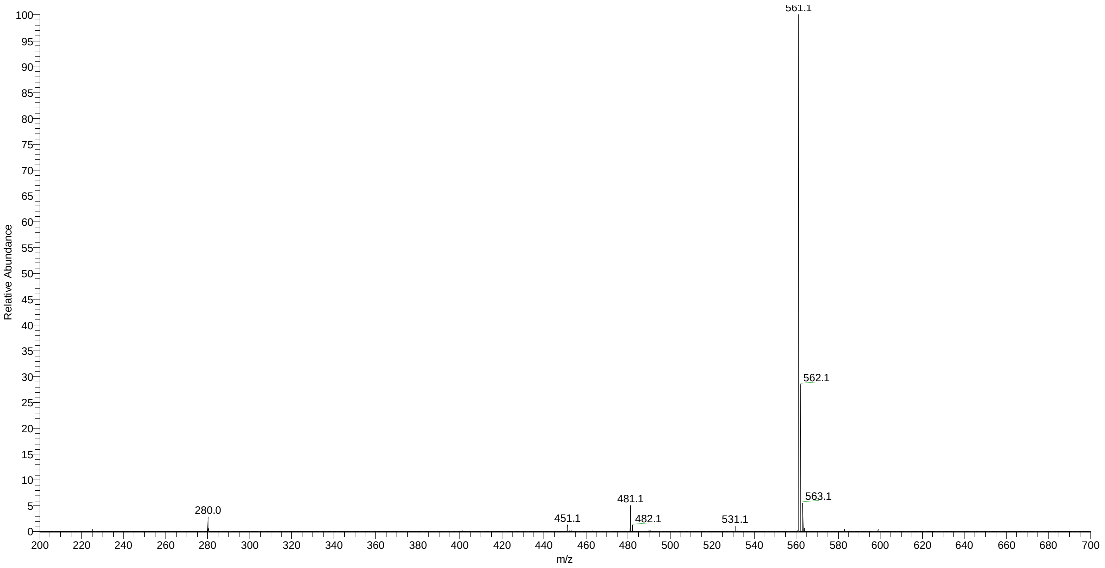

Figure S9. ESI-MS– spectrum of silychristin-19-*O*-sulfate.
([M – H]$^{-}$ *m/z* 561.1; [M – H – SO_3]$^{-}$, *m/z* 481.1)

Table S3. ^{13}C and ^{1}H NMR data of silychristin-19-*O*-sulfate.
(600.23 MHz for ^{1}H, 150.95 MHz for ^{13}C, DMSO-d_6, 30 °C)

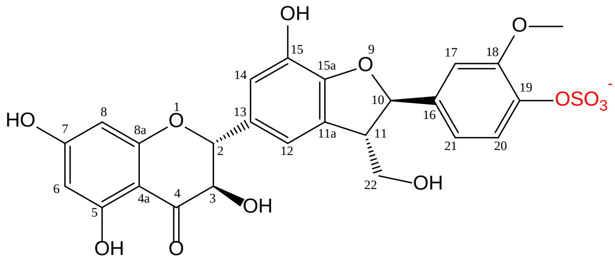

Atom	δ_C	m.	δ_H	n(H)	m.	*J* [Hz]
**2**	83.29	D	4.981	1	d	11.3
**3**	71.74	D	4.498	1	dd	11.3, 6.2
**4**	197.56	S	-	0		
**4a**	100.32	S	-	0		
**5**	163.36	S	-	0		
**6**	96.17	D	5.878	1	d	2.0
**7**	167.2[a]	S	-	0		
**8**	95.17	D	5.844	1	d	2.0
**8a**	162.56	S	-	0		
**10**	86.85	D	5.509	1	d	7.0
**11**	53.60	D	3.489	1	ddd	7.0, 6.8, 5.5
**11a**	128.96	S	-	0		
**12**	115.36	D	6.871	1	d	1.7
**13**	130.19	S	-	0		
**14**	115.78	D	6.831	1	d	1.7
**15**	140.79	S	-	0		
**15a**	147.11	S	-	0		
**16**	136.66	S	-	0		
**17**	110.68	D	6.997	1	d	2.0
**18**	150.55	S	-	0		
**19**	142.53	S	-	0		
**20**	120.93	D	7.438	1	d	8.3
**21**	117.74	D	6.882	1	dd	8.3, 2.0
**22**	62.99	T	3.659	1	ddd	10.9, 6.8, 5.7
			3.747	1	ddd	10.9, 5.5, 5.2
**3-OH**	-	-	5.707	1	d	6.2
**5-OH**	-	-	11.916	1	s	
**7-OH**	-	-	10.747[b]	1	br s	
**15-OH**	-	-	10.315[b]	1	s	
**18-OMe**	55.83	Q	3.737	3	s	
**22-OH**	-	-	5.010	1	dd	5.7, 5.2

[a] - HSQC readout; [b] - might be interchanged, m - multiplicity

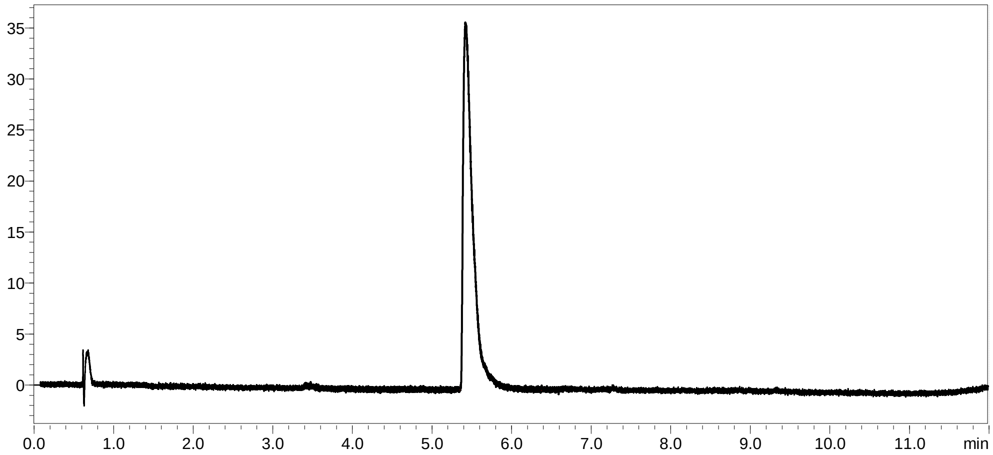

Figure S10. HPLC chromatogram of silychristin-19-*O*-sulfate.

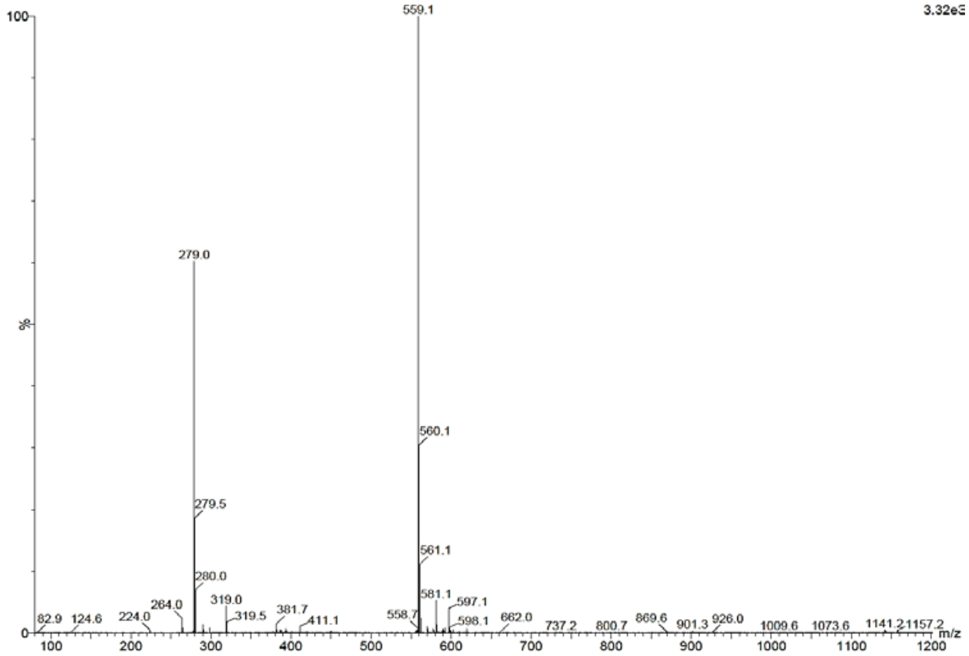

Figure S11. ESI-MS– spectrum of 2,3-dehydrosilychristin-19-*O*-sulfate ([M – H]$^{-}$, *m/z* 559.1; [M – 2H]$^{2-}$, *m/z* 279.0.)

Table S4. ^{13}C and ^{1}H NMR data of 2,3-dehydrosilychristin-19-*O*-sulfate. (600.23 MHz for ^{1}H, 150.95 MHz for ^{13}C, DMSO-d_6, 30 °C)

Atom	δ_C	m.	δ_H	n(H)	m.	*J* [Hz]
**2**	146.84	S		0		
**3**	135.84	S		0		
**4**	175.91	S		0		
**4a**	103.08	S		0		
**5**	160.77	S		0		
**6**	98.26	D	6.193	1	d	2
**7**	163.98	S		0		
**8**	93.44	D	6.420	1	d	2
**8a**	156.22	S		0		
**10**	87.30	D	5.602	1	d	6.6
**11**	53.27	D	3.578	1	m	
**11a**	129.74	S		0		
**12**	115.58	D	7.610	1	dd	1.8, 1.0
**13**	123.94	S		0		
**14**	116.00	D	7.647	1	dd	1.8, 0.4
**15**	140.98	S		0		
**15a**	148.76	S		0		
**16**	136.43	S		0		
**17**	110.72	D	7.007	1	d	2.1
**18**	150.61	S		0		
**19**	142.64	S		0		
**20**	121.01	D	7.457	1	d	8.3
**21**	117.78	D	6.894	1	ddd	8.3, 2.1, 0.4
**22**	63.01	T	3.774	1	m	
			3.722	1	m	
**3-OH**	-	-	9.347	1	s	
**5-OH**	-	-	12.457	1	s	
**7-OH**	-	-	10.757	1	s	
**15-OH O**	-	-	9.599	1	s	
**18-OMe**	55.87	Q	3.745	3	s	
**22-OH**	-	-	5.081	1	dd	ΣJ = 10.8

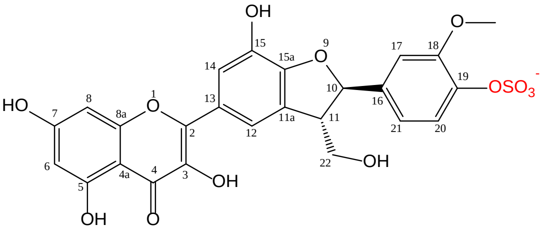

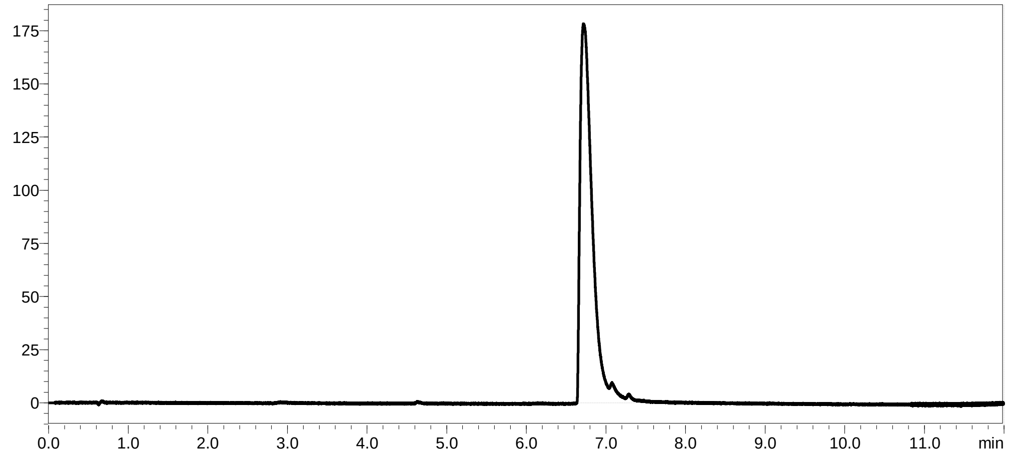

Figure S12. HPLC chromatogram of 2,3-dehydrosilychristin-19-*O*-sulfate.

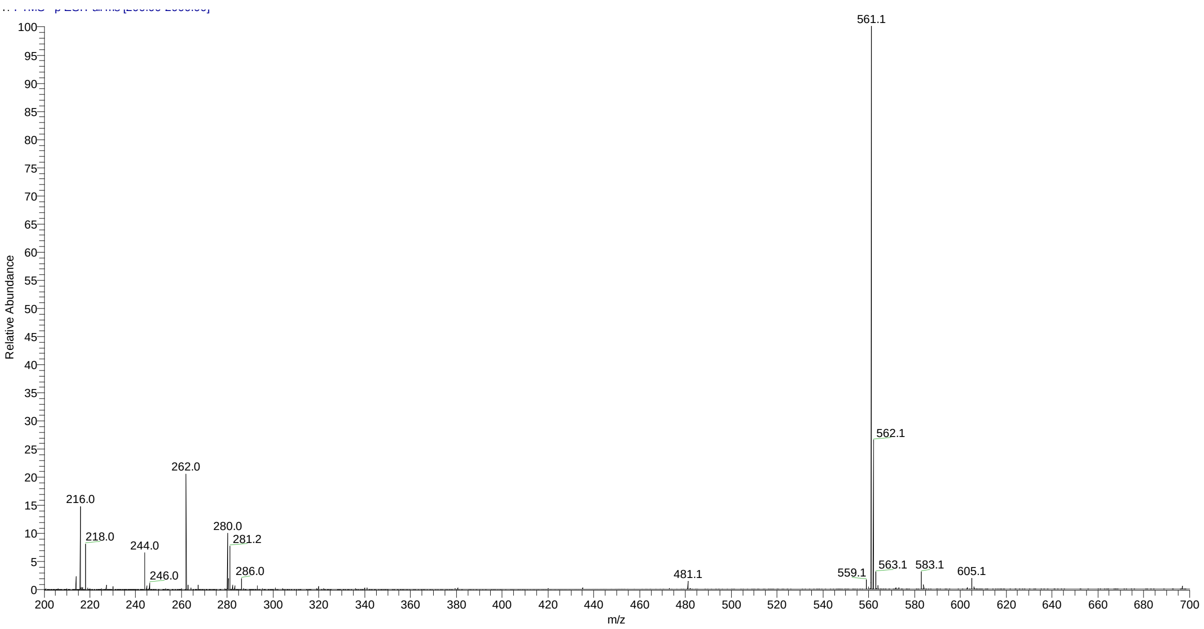

Figure S13. ESI-MS− spectrum of silydianin 19-*O*-sulfate.
([M – H]$^-$, *m/z* 561.1)

Table S5. ^{13}C and ^{1}H NMR data of silydianin 19-*O*-sulfate (600.23 MHz for ^{1}H, 150.95 MHz for ^{13}C, DMSO-d_6, 30 °C)

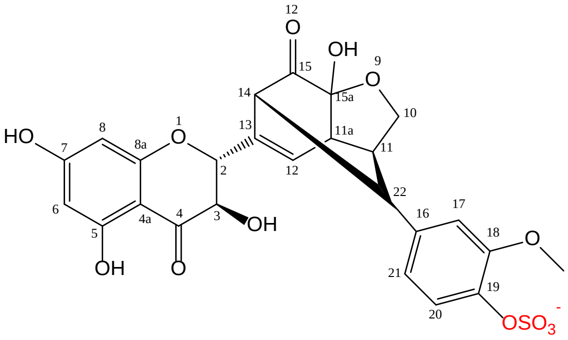

Atom	δ_C	m.	δ_H	n(H)	m.	*J* [Hz]
**2**	81.66	D	4.835	1	dd	10.9, 0.7
**3**	70.82	D	4.446	1	d	10.9
**4**	196.19	S	-	0		
**4a**	99.95	S	-	0		
**5**	163.38	S	-	0		
**6**	96.53	D	5.86[a]	1	m	
**7**	167.74	S	-	0		
**8**	95.34	D	5.85[a]	1	m	
**8a**	162.07	S	-	0		
**10**	72.79	T	4.143	1	dd	7.9, 3.3
			3.799	1	d	7.9
**11**	44.18	D	2.769	1	m	ΣJ = 9.2
**11a**	48.58	D	3.481	1	dd	4.2, 2.2
**12**	139.79	S	-	0		
**13**	124.39	D	6.101	1	dd	7.1, 2.0
**14**	53.15	D	3.231	1	dd	6.8, 2.9
**15**	201.76	S	-	0		
**15a**	96.81	S	-	0		
**16**	137.32	S	-	0		
**17**	112.94	D	6.809	1	d	2.1
**18**	150.11	S	-	0		
**19**	141.39	S	-	0		
**20**	120.58	D	7.340	1	d	8.4
**21**	119.71	D	6.673	1	dd	8.4, 2.1
**22**	46.24	D	3.356	1	m	ΣJ = 4.6
**3-OH**	-	-	5.788	1	br s	
**5-OH**	-	-	11.77	1	br s	
**7-OH**	-	-	n.d.			
**15a-OH**	-	-	n.d.			
**18-OMe**	55.60	Q	3.723	3	s	

[a] … HSQC readout
